# Which psychotherapy is most effective and acceptable in the treatment of adults with a (sub)clinical borderline personality disorder? A systematic review and network meta-analysis

**DOI:** 10.1017/S0033291723000685

**Published:** 2023-06

**Authors:** Kim Setkowski, Christina Palantza, Wouter van Ballegooijen, Renske Gilissen, Matthijs Oud, Ioana A. Cristea, Hisashi Noma, Toshi A. Furukawa, Arnoud Arntz, Anton J. L. M. van Balkom, Pim Cuijpers

**Affiliations:** 1 Research Department, 113 Suicide Prevention, Amsterdam, the Netherlands; 2 Department of Clinical, Neuro and Developmental Psychology, Vrije Universiteit, Amsterdam, the Netherlands; 3 Department of Psychiatry, Amsterdam UMC, VU University, Amsterdam Public Health research institute, and GGZinGeest Specialized Mental Health Care, Amsterdam, the Netherlands; 4 Department of Treatment, Care and Reintegration, Trimbos Institute, Utrecht, the Netherlands; 5 Department of Brain and Behavioral Sciences, University of Pavia, Pavia, Italy; 6 IRCCS Mondino Foundation, Pavia, Italy; 7 Department of Data Science, The Institute of Statistical Mathematics, Tokyo, Japan; 8 Department of Health Promotion and Human Behavior, Kyoto University Graduate School of Medicine/School of Public Health, Kyoto, Japan; 9 Department of Clinical Psychological Science, Maastricht University, Maastricht, the Netherlands; 10 Department of Clinical Psychology, University of Amsterdam, Amsterdam, the Netherlands

**Keywords:** Borderline personality disorder, mental healthcare, network meta-analysis, psychotherapy, suicidal behaviour

## Abstract

A broad range of psychotherapies have been proposed and evaluated in the treatment of borderline personality disorder (BPD), but the question which specific type of psychotherapy is most effective remains unanswered. In this study, two network meta-analyses (NMAs) were conducted investigating the comparative effectiveness of psychotherapies on (1) BPD severity and (2) suicidal behaviour (combined rate). Study drop-out was included as a secondary outcome. Six databases were searched until 21 January 2022, including RCTs on the efficacy of any psychotherapy in adults (⩾18 years) with a diagnosis of (sub)clinical BPD. Data were extracted using a predefined table format. PROSPERO ID:CRD42020175411. In our study, a total of 43 studies (*N* = 3273) were included. We found significant differences between several active comparisons in the treatment of (sub)clinical BPD, however, these findings were based on very few trials and should therefore be interpreted with caution. Some therapies were more efficacious compared to GT or TAU. Furthermore, some treatments more than halved the risk of attempted suicide and committed suicide (combined rate), reporting RRs around 0.5 or lower, however, these RRs were not statistically significantly better compared to other therapies or to TAU. Study drop-out significantly differed between some treatments. In conclusion, no single treatment seems to be the best choice to treat people with BPD compared to other treatments. Nevertheless, psychotherapies for BPD are perceived as first-line treatments, and should therefore be investigated further on their long-term effectiveness, preferably in head-to-head trials. DBT was the best connected treatment, providing solid evidence of its effectiveness.

## Introduction

Borderline personality disorder (BPD) is characterised by severe functional impairments, high levels of distress, and a high risk of suicide (Leichsenring, Leibing, Kruse, New, & Leweke, 2011; Skodol et al., 2002). Although research has found that suicide occurs in up to 10% of all people diagnosed with BPD (APA, 2001; Black, Blum, Pfohl, & Hale, 2004), lower rates have been reported (Gunderson et al., 2011; Temes, Frankenburg, Fitzmaurice, & Zanarini, 2019; Zanarini, Frankenburg, Reich, & Fitzmaurice, 2012), suggesting that these percentages might fluctuate across studies (Links, Boursiquot, & Links, 2021). The prevalence rate for attempted suicide, however, is much larger ranging from 55 to 85% (Links, Boursiquot, & Links, 2021). Although most people with BPD experience a high burden of disease, about 85% achieve remission (e.g. not fulfilling DSM-criteria) within 10 years (Gunderson et al., 2011; Zanarini et al., 2007). Evidence-based psychological treatments are perceived as first-line treatments for persons with BPD (Cristea et al., 2017; Oud, Arntz, Hermens, Verhoef, & Kendall, 2018; Storebo et al., 2020), although not one specific treatment is being favoured over the others. There is still an ongoing debate about the comparative efficacy of different types of treatments for adults with BPD.

So far, a few studies have provided meta-analytic evidence on the efficacy of psychotherapies for BPD. Firstly, a meta-analysis from 2017 (Cristea et al., 2017) investigated the efficacy of stand-alone therapies and add-on designs in the treatment of people with BPD, finding a significant improvement of BPD severity (*g* = 0.35) compared to TAU. They also found a significant decrease of suicide (attempts) and suicidal ideation (*g* = 0.41). A second review (Oud et al., 2018), examined the effectiveness of four BPD-specific treatments, finding a moderate significant effect size on BPD severity (*d* = 0.59) when compared to TAU. Their results on suicidal behaviour were inconclusive. A Cochrane review (Storebo et al., 2020) also found beneficial effects for various psychotherapies on BPD (*d* = 0.52) after pooling them together vis-á-vis TAU. They also found beneficial effects for various types of psychotherapies on suicide (attempts) RR = 0.27, 95% CI (0.11–0.67) compared to TAU. Recently, another meta-analysis was published (Stoffers-Winterling et al., 2022), evaluating the effects of stand-alone and add-on therapies for BPD. They found significant results for DBT (*d* = 0.54) and MBT (RR = 0.51) on self-harm *v.* TAU. A small, but significant effect for MBT on suicide-related outcomes was found (RR = 0.10). However, the authors did not make any comparisons among active treatments, nor were they able to include other specialised treatments such as ST and TFP, due to a lack of available trials. Still another meta-analysis (Rameckers et al., 2021) analysed pre-post changes of all design types, allowing comparisons of all treatments. ST was superior and TAU inferior to the average effect of all studies on BPD-severity. As to suicidality, TAU and CTBE were inferior, whereas ST and MBT were superior to the average treatment effect. We also noticed important discrepancies in the conclusions of all five reviews (Cristea et al., 2017; Oud et al., 2018; Rameckers et al., 2021; Stoffers-Winterling et al., 2022; Storebo et al., 2020). Despite the fact that these papers focus on a similar topic and do seem to have established the same PICO, they tend to differ in terms of their aims and scope, their methodological approach, and the trials they included.

There is not enough research to answer the question if one specific form of psychotherapy is more effective in treating BPD compared to others (Ellison, 2020; Leichsenring et al., 2011; Yeomans, Kenneth, & Meehan, 2012), because there are very few trials that compare two or more treatment types directly. Long-term outcomes of psychotherapies have also not yet been examined in a network meta-analysis (NMA). Two conventional meta-analyses (Oud et al., 2018; Storebo et al., 2020) are the only papers including head-to-head-trials, but both papers were not able to fully exploit the data, and compare different sets of psychotherapies that have not been compared directly in randomised controlled trials (RCTs) before. An NMA is a better approach than conventional meta-analyses, because it introduces a rank ordering in the selected psychotherapies, pooling evidence from both direct, and indirect comparisons (Rouse, Chaimani, & Li, 2017).

This is the first study to update previous reviews (Cristea et al., 2017; Oud et al., 2018; Storebo et al., 2020) by including additional RCTs and to elucidate the comparative efficacy of psychotherapies in adults with BPD through network meta-analyses (NMAs). In this paper, we also examined study drop-out as a secondary outcome measure.

## Methods

We followed PRISMA guidelines (Page et al., 2021) and PRISMA-NMA (Hutton et al., 2015) in conducting and reporting this systematic review and NMA.

### Study protocol and search strategy

PROSPERO ID: CRD42020175411. Changes to the protocol are described in online Supplementary Table S1. Six electronic databases (PsycINFO, PubMed, Embase, Scopus, the Cochrane Central Register of Controlled Trials and Web of Science) were systematically searched from inception to the 21^th^ of January 2022 (online Supplementary Table S1). We included papers written in English, Dutch, Greek and German according to the languages spoken by the authors.

### Study selection

The records were imported into Covidence Systematic Review Software for the screening process. After removal of duplicates, two independent assessors (KS and CP) screened the titles and abstracts. Both assessors conducted a full-text review of the remaining studies. The reference lists of studies included in the full-text review were also searched for relevant articles. In case of disagreement, consensus was reached by discussion with a third researcher (WvB).

### Eligibility criteria

Studies were eligible for inclusion if they used an RCT design testing the efficacy of psychotherapy, and if their study population consisted of adults (mean age of ⩾ 18 years) with a primary diagnosis of (sub)clinical BPD, assessed with a structured clinical interview according to ICD or DSM criteria. Comorbid disorders were not excluded, as long as studies primarily included adults with BPD. To meet the homogeneity assumption of the NMA, only studies performed in an outpatient setting were included. Web-based interventions were therefore not included in our study. Similar as to (Oud et al., 2018), we aimed to exclude interventions that did not have the potential to be delivered as a ‘complete therapy’ for people with BPD, but as an adjunct treatment instead, such as Systems Training for Emotional Predictability and Problem Solving, Emotion Regulation Training, or adjunctive Emotion Regulation Group Therapy.

### Definition of psychotherapy

Given the diversity of therapy orientations, we used an inclusive approach in selecting the psychotherapy and control conditions, by using the following definition: A treatment that is (1) based on psychological principles, (2) involves a trained therapist and a patient who is seeking help for a mental disorder (in this case BPD), problem, or complaint, (3) is intended by the therapist to be remedial for this disorder, problem, or complaint of the patient, and (4) is adapted or individualised for the particular patient and his or her disorder, problem, or complaint (Wampold, & Imel, 2015, p. 37).

### Interventions

Based on the expertise from one of the co-authors (AA), each specialised psychotherapy was classified into separate categories. The procedure of categorising each intervention in a separate node, is described in more detail in online Supplementary Table S2. A description of each type of treatment is presented in Table 1.

**Table 1.** Description for each type of psychotherapy classified into the nodes

Nodes	Description
Cognitive Behaviour Therapy (CBT)	In CBT, therapists focus on patients' dysfunctional thoughts and beliefs and their impact on current behaviour. This treatment is aimed at exploring, challenging and restructuring patients' dysfunctional beliefs. In addition to this cognitive work, behaviour therapy methods can be added to change dysfunctional behaviours. Teaching patients new methods of coping with stressful situations is an example of this. CBT involves homework assignments.
Community Treatment by Experts (CTBE)	CTBE is perceived as a special form of TAU [i.e. psychotherapy-as-usual provided by trained community experts with experience in treating people with BPD and/or personality disorders (PD)].
Dialectical Behaviour Therapy (DBT)	DBT was developed by Marsha Linehan and is based on the premise that emotional hyperreactivity and the use of dysfunctional emotion regulation strategies are central to BPD. DBT makes use of cognitive, behavioural and problem-solving interventions, including psychoeducation, problem solving and acceptance strategies, to help patients using more functional emotion regulation strategies. A dialectical stance is taken with respect to the need for change on the one hand, and the need for acceptance on the other hand. DBT also focuses on enhancing patients' motivation and engagement in treatment to prevent dropout. Treatment includes group skills training, individual therapy, and telephone contact (if needed).
Generic Treatments for BPD (GT)	GPM, SCM, CCT, and STM are classified as generic treatments for BPD. These interventions are either specifically developed for BPD, based on a model globally describing important developmental- and sustaining factors of BPD, or based on evidence based sophisticated forms of psychotherapy, adapted to BPD (e.g. CCT). All these treatments follow a global protocol, defining and optimising the therapeutic relationship, the techniques applied, and phases of treatment. In general, approach and technique allow a personalisation based on individual needs of the patient. (Thus, both the model and the techniques are less specific than those of specialised therapies, such as DBT, MBT, ST, and TFP, but more specific than general treatment approaches). Therapy needs to be delivered by experts in BPD treatment.
Interpersonal Psychotherapy (IPT)	Therapy is an adaptation of IPT (originally developed to treat major depression), whereby BPD is treated as a mood-inflicted disorder (such as dysthymic disorder) but with outburst of anger. As with IPT for depression, the focus is on interpersonal problems, with therapists helping patients to resolve them in a nondirective way. For IPT-BPD, length of treatment is prolonged. Therapy includes telephone contact with a clinician to prevent crises and improve therapeutic alliance, and psychoeducation for family members of patients.
Mentalisation-Based Treatment (MBT)	MBT, developed by Anthony Bateman and Peter Fonagy, is focused on understanding the process of one's own, and others' actions in terms of mental states (such as beliefs and intentions). MBT is based on a psychoanalytic model with cognitive therapeutic elements, aiming to stimulate patients to mentalise, and to develop more adaptive interpersonal behaviours. Psychoeducation is delivered. Attachment theory is explicitly taken into account in therapeutic attitude and techniques. Focus of treatment is the relationship with the therapist and other people, including other patients when a group format is used, whereby therapists seek to improve a patients' capacity to mentalise, as well as to identify their affective experience. Mentalising functional analysis, stop-stand-and-rewind techniques are used during treatment.
Mixed therapeutic techniques (mixed)	Psychotherapies were classified within the mixed category, if they used a combination multiple techniques from already existing interventions, such as ST, DBT, MBT or PDP.
Psychodynamic Psychotherapy (PDP)	PDP primarily focuses on improving the patients' understanding, awareness, and insight about (unconscious) intrapsychic conflicts and their interpersonal consequences. Therapists focus on patients' childhood experiences, historical relationships, and the impact these have on patients' present intra- and interpersonal functioning. Therapists try to strengthen patients' self image and self-reflective behaviour, and focus on maturation of maladaptive defence- and coping strategies. Elements of emotion regulation, identification of dysfunctional impulses, and reflecting on self- and others behaviour are also included. This category includes different forms of PDP, ranging from primarily focusing of increasing awareness of intrapsychic conflicts with therapists taking a neutral stance, to forms where the therapist is more supportive and more focused on encouraging the development of mature forms of defence mechanisms and relationships. However, the purely supportive forms are not included here, but with the Generic Treatments. Thus, all PDP forms make use of clarification, confrontation and interpretation as interventions.
Schema Therapy (ST)	ST is an integrative therapy in a cognitive framework developed by Jeffrey Young, that works with schema modes (patterns of thinking, feeling, and behaving). Schema modes result from activation of dysfunctional schemas and represent the specific states patients can be in. Dysfunctional schemas are theorised to result from early experiences during which core emotional needs were not met. Therapists aim to reduce dysfunctional schema modes and increase constructive ones. ST primarily uses cognitive, experiential and behavioural interventions. The therapeutic relationship offers corrective experiences via ‘limited reparenting’. Psychoeducation and processing of adverse childhood experiences that lie at the root of the development of dysfunctional schemas are important components.
Transference-Focused Therapy (TFP)	TFP has been developed by Otto Kernberg as an adaptation of psychodynamic psychotherapy suitable for BPD. According to Kernberg, the BPD core deficit is the person's disordered and incoherent mental representations of self and others. These representations are ‘split’, or organised in all-positive or all-negative parts (polarity), whilst the patient is not aware of this splitting. This splitting experience makes it difficult for someone to regulate emotions and behaviours properly. The manifestation of borderline pathology within the therapeutic relationship is explored with among other techniques confrontation and interpretation. Thus the therapists try to reduce the splitting of representations, pointing out conflicting elements of the patients' views of self or others, in order to achieve more realistic views. The focus of TFP is primarily on the here and now, and not on the patient's past.

### Primary and secondary outcome measures

The primary outcomes were (1) overall BPD severity and (2) suicidal behaviour defined as the combined rate of suicide attempts (i.e. reported by participants via questionnaires or during interviews) and death by suicide (i.e. identified from medical records). Effect sizes were based on the number of participants in the intervention and control condition who had engaged in suicidal behaviour. We accepted any validated clinician or self-rated instrument for overall BPD severity. If a study used more than one scale, we chose the scale that was most frequently used by other included studies. In case overall BPD severity was not measured, single BPD symptoms were extracted, assessed with a valid clinician or self-rated instrument. If trials measured more than one single BPD symptom, we selected those that were measured by most included studies, since it is not possible to use composite scores in NMAs. Study drop-out was measured as a secondary outcome and operationalised as study drop-out for any reason, and not as treatment drop-out, given that the latter one is often defined differently across trials, making it difficult to statistically pool and interpret the results.

### Data extraction and quality assessment

Risk of bias (RoB) for all trials was assessed independently by two assessors (KS and CP) using the Revised Cochrane risk-of-bias tool 2.0 for randomised trials (Higgins et al., 2019). We reported the overall RoB score for both outcomes separately in the online Supplementary Table S3 as well. The data were independently extracted by the two assessors (KS & CP). If reported, we extracted the data from the intention-to-treat analysis (ITT) in each included study.

### Data analysis

In this study, two NMAs were conducted. The first NMA focused on examining the comparative efficacy for psychotherapies on BPD symptom severity, while the second NMA aimed at suicidal behaviour. Study drop-out was included as a secondary outcome. Not all eligible studies (*N* = 43) were included in both NMAs, because not all RCTs measured both primary outcomes simultaneously within their trial. For continuous outcomes, standardised mean difference (SMD) was calculated between each of the contrasts based on mean, s.d. and number of participants for the conditions. Dichotomous outcomes were reported as Relative Risks (RRs) with 95% confidence intervals (CIs). RRs for suicidal behaviour were calculated as a measure of ratio of the probability of participants engaging in suicidal behaviour. For study drop-out, RRs was calculated as a measure of ratio of the probability of events.

Firstly, we conducted random-effects pairwise meta-analyses for every treatment comparison, given the expected clinical and methodological heterogeneity of treatment effects between studies. We calculated the *I*^2^ statistic as an indicator of heterogeneity (%) with 95% CIs. Publication bias was assessed by Egger's test to investigate the asymmetry of funnel plot. To assess the overall publication bias on the network, we used comparison-adjusted funnel plots to investigate whether biases of the results from active treatments comparing with control conditions (TAU) existed.

Secondly, we implemented NMA using the contrast-based, random-effects multivariate meta-analysis model (Salanti, 2012; White, Barrett, Jackson, & Higgins, 2012). We made graphical illustrations of the network evidence base by network plots (Chaimani, Higgins, Mavridis, Spyridonos, & Salanti, 2013). We conducted synthesis analyses using the NMA model for the comparative efficacy and study drop-out. Comparative SMDs and RRs were reported with their 95% CIs and 95% prediction intervals (PrI). The PrI illustrates the prediction interval covering the true treatment effect in a future study with 95% probability. To evaluate influences of individual comparisons for the entire network, contribution plots were developed. To assess the ranking of the treatments, we used the surface under the cumulative ranking curve (SUCRA) (Salanti, Ades, & Ioannidis, 2011). The larger the surface below the SUCRA, the more efficacious the treatment will be. SUCRA would be 1 when a treatment is certain to be the best, and 0 when a treatment is certain to be the worst.

To examine the transitivity assumption, we conducted the local and global inconsistency tests (Rouse et al., 2017). The local inconsistency tests correspond to a loop specific approach that investigates local inconsistency, and we conducted the inconsistency test for all the triangular or quadratic loops in the network (Bucher, Guyatt, Griffith, & Walter, 1997). Besides, the global inconsistency test is a goodness-of-fit test using the design-by-treatment interaction model of Higgins et al. (Higgins et al. 2012).

We conducted a series of five sensitivity analyses to examine the robustness of the results: (1) one including studies using interventions with all four DBT components (categorised as ‘full DBT’), (2) another one only including trials reporting on suicides and suicide attempts, and (3) one analysis only including studies reporting overall BPD severity. Lastly, (4) a sensitivity analysis including studies only categorised as low RoB, (5) and one sensitivity analysis only including studies using an individual + group format. Statistical analyses were conducted using Stata version 16, and mvmeta (White, 2011; White et al., 2012), network packages (White, 2015), network graphs (Chaimani & Salanti, 2015), and heterogi module (Orsini, Bottai, Higgins, & Buchan, 2006).

## Results

### Selection, inclusion and characteristics of studies

**Fig. 1.**
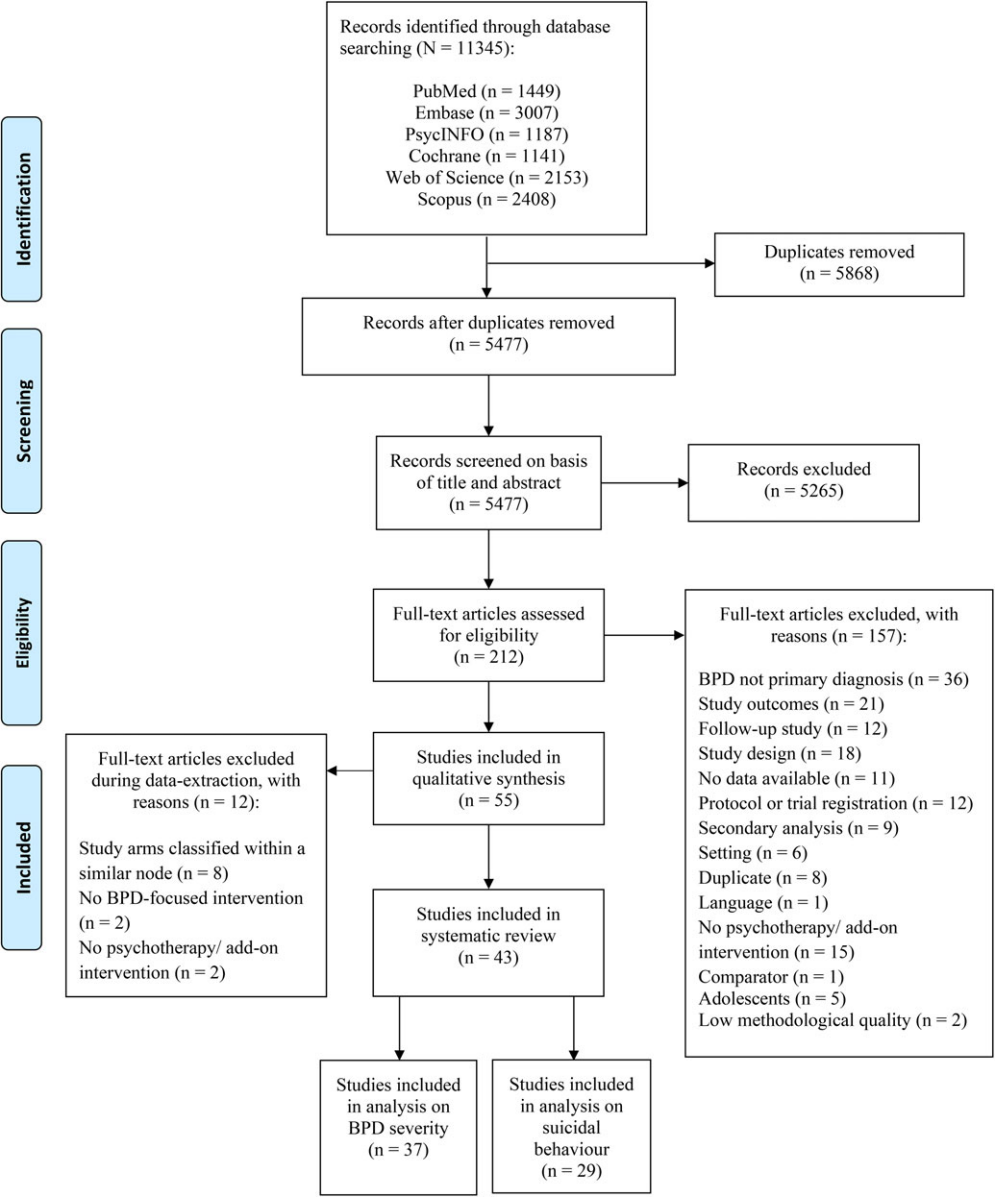
Flowchart for inclusion of studies.

A total of 11.345 records were identified through database searching (Fig. 1). After removal of duplicates 5477 unique titles were independently screened. We retrieved 212 full-text papers for further consideration and excluded 157 studies, resulting in a total of 55 papers (*N* = 4044). The PRISMA flowchart is illustrated in Fig. 1.

A total of eight studies were excluded because their study arms were classified into the same node (Chanen et al., 2022; Elices et al., 2016; Farrés et al., 2019; Harned, Korslund, & Linehan, 2014; Laursen et al., 2021; Linehan et al., 2015; Smits et al., 2020; Wolf et al., 2011). Two studies (Gleeson et al., 2012; Robinson et al., 2016) were excluded because their study arm(s) were primarily focused on a different psychiatric disorder, such as eating disorders and psychosis. One study (Feliu-Soler et al., 2017) was excluded because their intervention was developed as a training, rather than a psychotherapeutic treatment (Wampold, & Imel, 2015, p. 37). We also removed one pilot study (Morton, Snowdon, Gopold, & Guymer, 2012), because their intervention was not delivered as a full course treatment, but rather as a brief-adjunct intervention, only meant to be provided as an addition to a current treatment (i.e. Gratz et al., 2006). This resulted in a total of 43 studies (*n* = 3273), including eleven conditions (psychotherapies and control conditions) that were used for the statistical analyses. The 43 studies included 9 PDP, 5 GT, 5 CBT, 19 DBT, 5 MBT, 3 ST, 3 TFP, 2 IPT, 6 CTBE arms as well as 22 TAU arms (Table 2). We also classified 10 arms into a category of interventions using multiple components of different treatments, called ‘mixed interventions’ (mixed). Furthermore, a total of ten studies did not perform an ITT-analysis (Bellino, Rinaldi, & Bogetto, 2010; Bozzatello & Bellino, 2020; Carlyle et al., 2020; Crawford et al., 2020; Farrell, Shaw, & Webber, 2009; Jorgensen et al., 2013; Koons et al., 2001; Linehan, Armstrong, Suarez, Allmon, & Heard, 1991; Stanley, 2017; Weinberg, Gunderson, Hennen, & Cutter, 2006). Figure 1 shows these 43 RCTs were included in the statistical analyses (*N* = 3273).

**Table 2.** Table with selected characteristics of included studies (*N* = 43)

		Sample size (*N_r_*)		Female *N* (%)	Age M (s.d.)*	Treatment				Outcomes		Follow-up (weeks)		Nodes	
	Author, country	Intervention	Control			Format	Modality	No sessions	Duration (weeks)**	BPD severity	Suicide	BPD severity	Suicide	Intervention	Control
1	Amianto, 2011 (Italy)	18	17	17 (49)	39.6	Ind	Ftf	40	52^a^	CGI	CGI-BPD	26, 52 wks	26, 52 wks	PDP	GT
2	Andreasson, 2016 (Denmark)	57	51	80 (74)	31.7 (12.7)	Ind & Grp	Ftf	32	28^b^	ZAN-BPD	count	not measured	not measured	DBT	mixed
3	Andreoli, 2016 (Switzerland)	140	30	120 (84)	31.9 (10.1)	Ind	Ftf	26	13	not measured	count	not measured	not measured	mixed	CTBE
4	Bateman, 2009 (UK)	71	63	107 (80)	31.1	Ind & Grp	Ftf	140	78	IIP	count	not measured	not measured	MBT	GT
5	Bellino, 2010 (Italy)	27	28	35 (67)	26.0	Ind	Ftf	32	32	BPD-SI	BPD-SI^c^	26, 52, 104 wks	25, 52, 104 wks[Table-fn tfn2_7]	IPT	TAU
6	Bohus, 2020	103	97	200 (100)	36.3 (11.1)	Ind & Grp	Ftf	48	65	BSL-23	count	not measured	not measured	mixed	CBT^1^
7	Bozzatello, 2020 (Italy)	22	21	24 (67)	35.4 (14.7)	Ind	Ftf (+Tel)	42	40	BPD-SI	SHI^3^	not measured	not measured	IPT	TAU
8	Carlyle, 2020	38	34	71 (99)	32.0	Ind & Grp	Ftf	156	78	not measured	count	not measured	not measured	MBT	GT
9	Carter, 2010	38	35	73 (100)	24.5 (6.1)	Ind & Grp	Ftf	52	26	WHOQOL-BREF	PHI-2	not measured	not measured	DBT	TAU
10	Clarkin, 2007 (USA)	31	30	83 (92)	30.9 (7.9)	Ind & Grp	Ftf	104	52	BIS	oas-m- suicidality	not measured	not measured	TFP	DBT
			29												PDP
11	Cottraux, 2009 (France)	33	32	50 (77)	33.5	Ind	Ftf	36	52	Eysenck-IVE	count	52 wks	52 wks	CBT	GT
12	Crawford, 2020 (UK)	33	30	43 (68)	36.3	Ind	Ftf	6-10	24	not measured	SNH-SPM	not measured	not measured	mixed	TAU
13	Davidson, 2006 (UK)	54	52	Not reported	Not reported	Ind	Ftf	27	52	IIP-32	ADSHI	52 wks, 260 wks	52 wks, 260 wks	CBT	TAU
14	Dixon-Gordon, 2015 (USA)	13	6	19 (100)	34.5 (11.83)	Grp	Ftf	7	7	PAI-BOR	DSHI^3^	7 wks	7 wks[Table-fn tfn2_7]	DBT	TAU
15	Doering, 2010 (Germany)	52	52	104 (100)	27.3	Ind	Ftf	104	52	DSM-IV for BPD	CISSB	not measured	not measured	TFP	CTBE
16	Farrell, 2009 (USA)	16	16	32 (100)	35.6	Grp	Ftf	30	35	DIB-R	not measured	26 wks	not measured	ST	TAU
17	Feigenbaum, 2012 (UK)	25	16	30 (73)	35.1	Ind & Grp	Ftf (+Tel)	104	52	STAXI	SASII	not measured	not measured	DBT	CTBE
18	Giesen-Bloo, 2006 (the Netherlands)	45	43	80 (93)	30.6	Ind	Ftf	312	156	BPDSI-IV	BPDSI-IV	not measured	not measured	TFP	ST
19	Gregory, 2008 (USA)	15	15	24 (80)	28.7	Ind	Ftf	52	52	BEST-BPD	LPC	78 wks	not measured	PDP	TAU
20	Herpertz, 2020	30	29	38 (64.4)	31.5	Ind & Grp	Ftf	13	6-7	STAXI	oas-m-suicidality	26 wks	26 wks	mixed	TAU
21	Hilden, 2020	28	14	35 (83)	29.7	Grp	Ftf	20	20	BSL-23	not measured	not measured	not measured	ST	TAU
22	Jorgensen, 2013 (Denmark)	74	37	106 (95)	29.1	Ind & Grp	Ftf	208	104	SCID-BPD	not measured	182 wks	not measured	MBT	PDP
23	Koons, 2001 (USA)	14	14	28 (100)	35.0	Ind & Grp	Ftf	52	26	DSM-IV for BPD	PHI	not measured	not measured	DBT	TAU
24	Kredlow, 2017a	15	12	26 (96)	45.7 (9.6)	Ind	Ftf	3	3	SCID-II-BPD	not measured	26, 52 wks	not measured	CBT^1^	TAU
25	Laurenssen, 2018 (the Netherlands)	54	41	75 (79)	34.0	Ind & Grp	Ftf	Not reported^2^	78	BPDSI	SSHI (not reported)	not measured	not measured	MBT	CTBE
26	Leppänen, 2015 (Finland)	24	47	44 (86)	32.1	Ind & Grp	Ftf	92	52	BPDSI-IV	BPDSI-IV^c^	not measured	not measured	mixed	TAU
27	Lin, 2019 (Taiwan)	42	40	72 (88)	20.4	Grp	Ftf	8	8	BPD-FS	CMSADS-L	12, 24 wks	12, 24 wks	DBT	CBT
28	Linehan, 1991 (USA)	30	31	46 (100)	Not reported	Ind & Grp	Ftf ( + Tel)	104	52	not measured	count	not measured	not measured	DBT	TAU
29	Linehan, 2006 (USA)	60	51	101 (100)	29.3 (7.5)	Ind & Grp	Ftf ( + Tel)	104	52	STAXI	count	52 wks	52 wks	DBT	CTBE
30	Majdara, 2019 (Iran)	15	15	18 (60)	27.3	Ind (for intervention) & Grp (for control)	Ftf	52	52	BEST-BPD	not measured	13 wks	not measured	PDP	TAU
31	McMain, 2009 (Canada)	90	90	155 (86.1)	30.4 (9.9)	Ind & Grp	Ftf ( + Tel)	104	52	ZAN-BPD	SASII	26, 52, 78, 104 wks	26, 52, 78 104 wks	DBT	PDP
32	McMain, 2017 (USA)	42	42	66 (79)	29.7 (8.6)	Grp	Ftf	20	20	BSL-23	count	12 wks	not measured	DBT	TAU
33	Philips, 2018 (Sweden)	24	22	37 (80)	36.7 (9.6)	Ind & Grp	Ftf	63[Table-fn tfn2_13]^d^	78	BPDSI-IV	count	not measured	not measured	MBT	TAU
34	Pistorello, 2012 (USA)	31	32	51 (81)	20.9 (1.9)	Ind & Grp	Ftf ( + Tel)	60-104	52[Table-fn tfn2_14]^e^	SCID-BPD	SBQ	26 wks	26 wks	DBT	CTBE
35	Priebe, 2012 (UK)	40	40	70 (87.5)	32.2 (10.8)	Ind & Grp	Ftf ( + Tel)	104	52	ZAN-BPD	count (days of self-harm)^3^	unclear^4^	26 wks[Table-fn tfn2_7]	DBT	TAU
36	Reneses, 2013 (Spain)	25	28	31 (70)	33.8 (7.5)	Ind	Ftf	20	26[Table-fn tfn2_15]^f^	ZAN-BPD	ZAN-BPD-suicidality	26, 52 wks^5^	not measured	PDP	TAU
37	Soler, 2009 (Spain)	29	30	49 (83)	29.2	Grp	Ftf	13	13	CGI-BPD	CGI-BPD	not measured	not measured	DBT	PDP
38	Turner, 2000 (USA)	12	12	19 (79)	22.0	Ind & Grp	Ftf	49-84	52	IRIRS	count^3^	not measured	not measured	DBT	GT
39	Verheul, 2003 (the Netherlands)	31	33	58 (100)	34.9 (7.7)	Ind & Grp	Ftf	104	52	BPDSI-IV (impulsivity)	BPDSI	not measured	not measured	DBT	TAU
40	Visintini, 2020 (Italy)	41	54	81 (85)	26.2 (7.0)	Ind & Grp	Ftf	260	52	BIS-11	SHI-22^3^	not measured	not measured	DBT	mixed
41	Stanley, 2017 (USA)	42	42	77 (91.7)	29.3 (9.4)	Ind & Grp	Ftf ( + Tel)	52	26	not measured	CSHI	not measured	not measured	DBT	TAU
42	Walton, 2020	83	83	125 (77)	26.6 (7.8)	Ind & Grp	Ftf ( + Tel)	121	61	BPDSI-IV	SASI-C^3^	not measured	not measured	DBT	PDP
43	Weinberg, 2006 (USA)	15	15	30 (100)	28.2	Ind	Ftf	6	6-8	not measured	PHI	not measured	26 wks	mixed	TAU

Tel, phone coaching; ind, individual; grp, group; ftf, face-to-face; PDP, psychodynamic psychotherapy; GT, generic treatments for BPD; IPT, interpersonal psychotherapy; CTBE, community treatment by experts; DBT, dialectical behaviour therapy; CBT, cognitive behavioural therapy; MBT, mentalisation based therapy; TFP, transference-focused therapy; ST, schema therapy; mixed, mixed approaches; TAU, treatment-as-usual; CGI/CGI-BPD, clinical global impression, borderline personality disorder; ZAN-BPD, Zanarini scale for BPD; ZAN-BPD-suicidality, Zanarini scale for BPD – suicidality subscale; IIP/IIP-32, inventory of interpersonal problems; BPDSI/BPDSI-IV, borderline personality disorder severity index; BSL-23, borderline symptom list; WHOQOL-BREF, world health organization (who) quality of life-bref version; BIS/BIS-11, barratt impulsiveness scale; Eysenck-IVE, Eysenck impulsivity venturesomeness empathy inventory; PAI-BOR, personality assessment inventory- borderline features scale; DSM-IV-BPD, DSM diagnostic criteria for borderline personality disorder; DIB-R, diagnostic interview for borderline personality disorders revised; STAXI, state trait anger expression inventory; BEST-BPD, borderline evaluation of severity over time; SBQ, Suicidal Behaviors Questionnaire; SCID-BPD/SCID-II-BPD, structured clinical interview for dsm-iv personality disorders, borderline personality disorder; BPD-FS, borderline personality disorder features scale; IRIRS, independent rater impulsiveness rating scale; ADSHI/DSHI/SHI/SSHI, adapted version (deliberate) self-harm inventory; SHI-22, self-harm inventory-22; PHI/PHI-2, parasuicide history interview; oas-m-suicidality, overt aggression scale modified for suicidality; SNH-SPM, suicidal thoughts – national household survey of psychiatric morbidity; CISSB, cornell interview for suicidal and self-harming behaviour – self report; SASII, suicide attempt self-injury interview; LPC, lifetime parasuicide count; CMSADS-L, chine version of the modified schedule of affective disorders and schizophrenia-lifetime; CSHI, columbia suicide history interview; SASI-C, suicide attempt and self-injury count.

*N_r_* = number of participants randomised (reported for each condition separately).

* If studies did not report M age for total sample, a weighted average was calculated manually (without reporting s.d. in the Table).

** Treatment duration defined as number of weeks at post-test after baseline.

1 CBT with trauma-focused interventions.

2 The intervention was delivered 5 days/ w, 6 h/day for a total duration of 390 days.

3 The outcome was not included in the statistical analyses because it primarily measured the number of events/number of days engaging in suicidal behaviour.

4 The authors did perform a 6-month follow-up, but it was not reported whether bpd severity was monitored during follow-up or not.

5 Authors performed a follow-up study, using single BPD symptoms as outcome.

a Last session of treatment took place after 40 weeks and post-test at 52 weeks.

b Last session of treatment took place after 16 weeks and post-test at 28 weeks.

c Instrument used a scale to measure frequency of suicidal events as an outcome (not included in statistical analyses).

d Represents average number of sessions.

e Intervention duration was 7 to 12 months with post-assessment at 12 months.

f Last session of treatment took place after 20 weeks and post-test at 26 weeks.

The RCTs (*n* = 43) were published between 1991 and 2020. Sample sizes ranged from 19 to 200 participants per trial. A total of 2630 participants were female (80.4%). The mean age of participants ranged from 20.4–45.7 years.

Thirteen studies used an individual format and six studies delivered their treatment as a group format. The majority of studies combined both formats (*n* = 23). Most interventions were delivered face-to-face (*n* = 34), but nine studies added phone coaching to their face-to-face treatments. The study of Majdara et al. (2019) used different formats for each study arm: intervention was delivered in individual format, and the control condition was executed in group format. The number of sessions ranged widely from 3 sessions to a maximum of 312 sessions (median = 52.0). If studies reported a range for number of sessions, we used the highest number to calculate the median. One study did not report the number of sessions, but they delivered the intervention under study 5 days a week, for a total duration of 390 days (Laurenssen et al., 2018). The mean duration of all treatments (post-assessment after baseline) was 45 weeks (range: 3–156 weeks). If studies reported a range for treatment duration, we used the highest number to calculate the average treatment duration across included studies. Patient characteristics of the included studies (*n* = 43) are reported in Table 2.

### Methodological quality

From the 43 included RCTs, results from RoB assessment are illustrated in online Supplementary Table S3.

### BPD Symptom severity

#### Pairwise meta-analyses

We first conducted random-effects pairwise meta-analyses for every treatment comparison. Results are shown in online Supplementary Table S4.

#### Network plot

**Fig. 2.**
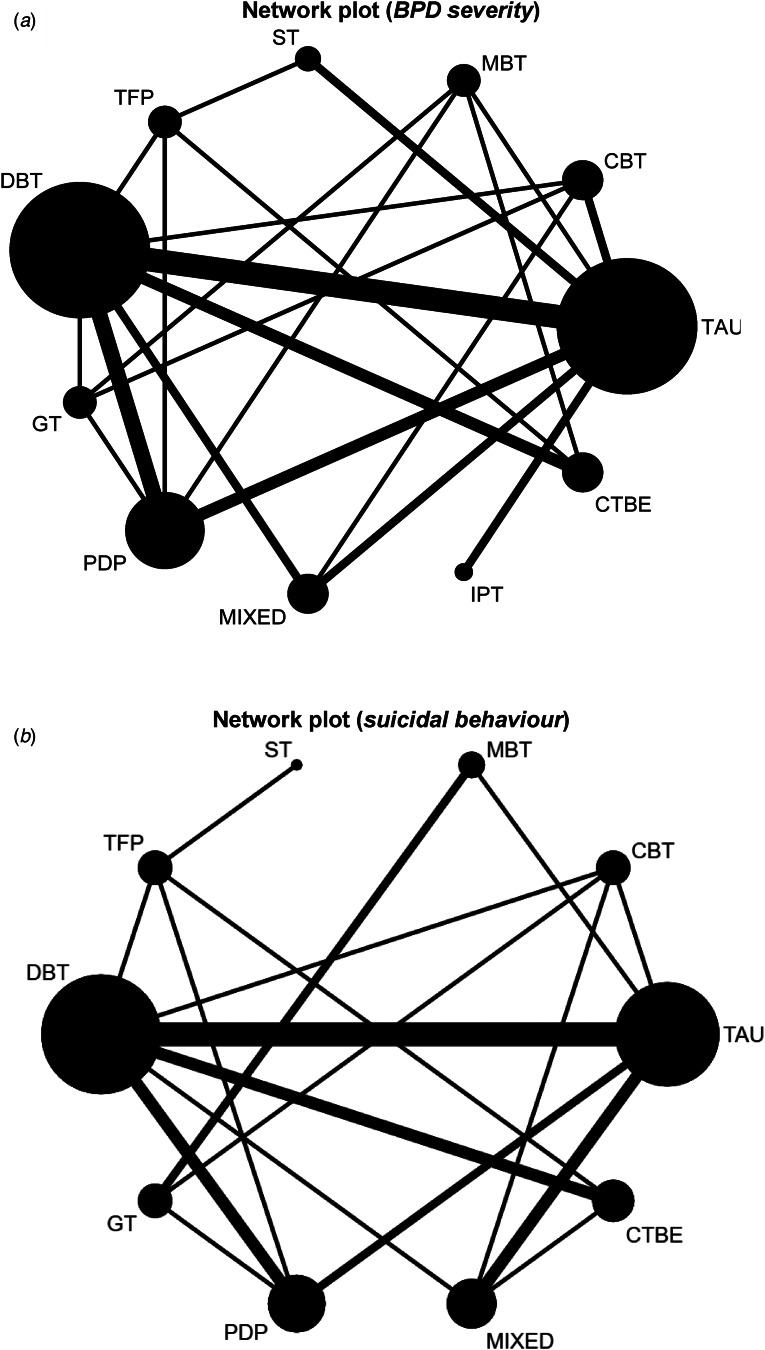
(*a*) Network plot for the efficacy of psychotherapies on BPD severity. The nodes and edges are weighted according to the number of participants (*N* = 2793) from 37 studies. MBT, mentalisation based therapy; CBT, cognitive behavioural therapy; TAU, treatment-as-usual; IPT, interpersonal psychotherapy; CTBE, community treatment by experts; PDP, psychodynamic psychotherapy; GT, generic treatments for BPD; DBT, dialectical behaviour therapy; TFP, transference-focused therapy; ST, schema therapy; mixed, mixed approaches/therapeutic techniques. (*b*) Network plot for the efficacy of psychotherapies on suicidal behaviour. The nodes and edges are weighted according to the number of participants (*N* = 2383) and comparisons from 29 studies. CBT, cognitive behavioural therapy; TAU, treatment-as-usual; PDP, psychodynamic psychotherapy; CTBE, community treatment by experts; GT, generic treatments for BPD; DBT, dialectical behaviour therapy; mixed, mixed approaches/ therapeutic techniques; TFP, transference-focused therapy; ST, schema therapy.

Of these 43 RCTs, a network plot was created for 37 studies (*n* = 2793), including 11 nodes: MBT (*n* = 223), CBT (*n* = 239), TAU (*n* = 469), CTBE (*n* = 192), IPT (*n* = 49), mixed (*n* = 262), PDP (*n* = 342), GT (*n* = 124), DBT (*n* = 678), TFP (*n* = 128), and ST (*n* = 87) as illustrated in Fig. 2a. Six studies were excluded in this NMA because they did not measure BPD severity but only measured suicidal behaviour (Andreoli et al., 2016; Carlyle et al., 2020; Crawford et al., 2020; Linehan et al., 1991; Stanley, 2017; Weinberg et al., 2006). Data was not provided by the authors after contacting them. From the 37 RCTs remaining, data on single BPD symptoms were extracted from 11 studies (Bateman & Fonagy, 2009; Carter, Willcox, Lewin, Conrad, & Bendit, 2010; Clarkin, Levy, Lenzenweger, & Kernberg, 2007; Cottraux et al., 2009; Davidson et al., 2006; Feigenbaum et al., 2012; Herpertz et al., 2020; Linehan et al., 2006; Turner, 2000; Verheul et al., 2003; Visintini, Roder, Gaj, & Maffei, 2020). Although impulsivity and anger were both equally often measured, impulsivity was most of the times assessed with a complete and valid scale, and therefore primarily extracted from studies (Clarkin et al., 2007; Cottraux et al., 2009; Turner, 2000; Verheul et al., 2003; Visintini et al., 2020), followed by anger (Clarkin et al., 2007; Feigenbaum et al., 2012; Herpertz et al., 2020; Linehan et al., 2006; Turner, 2000), and lastly, interpersonal problems (Bateman & Fonagy, 2009; Carter et al., 2010; Davidson et al., 2006). As illustrated in the network plot, the most examined comparisons were between dialectical behaviour therapy (DBT) and TAU (*N_c_*_omparisons_ = 6). Almost all nodes included at least three or more studies (except for IPT node) and were well connected, meaning that each node was at least connected to two other nodes within the network (except for IPT-node). The contribution plots show the degrees of contributions from the direct comparison evidence for the mixed and indirect estimates (online Supplementary Table S5a).

#### Network meta-analysis

The main results of the NMA are presented in Table 3. In terms of effectiveness, there was evidence that DBT (0.42, 95% CI 0.11–0.73) and ST (1.14, 95% CI 0.48–1.80) were more efficacious than TAU. MBT was borderland significant compared to TAU (0.54, 95% CI −0.02 to 1.10). ST also beat CTBE (1.01, 95% CI 0.25–1.77). These treatments were also more efficacious than GT, and ST was further shown to be more efficacious than CBT (0.90, 95% CI 0.12–1.69), PDP (0.76, 95% CI 0.04–1.48), and DBT (0.72, 95% CI 0.03–1.41). All the other comparisons had too wide CIs to allow meaningful inferences, making it more difficult to interpret the results (Table 3). We were unable to perform an NMA on long-term effects, because not enough studies were available (*n* = 17 (follow-up studies measuring bpd severity)) (Table 2), reporting large differences in follow-up outcomes between trials (ranging from 7 weeks to 260 weeks).

**Table 3.** Relative effect sizes of efficacy (SMD) for psychotherapies on BPD at post-treatment according to network meta-analysis

TAU										
0.13 (−0.38, 0.65)	CTBE									
0.52 (−0.20, 1.23)	0.39 (−0.50, 1.27)	IPT								
0.38 (−0.07, 0.82)	0.25 (−0.37, 0.86)	−0.14 (−0.98, 0.70)	Mixed							
0.38 (−0.00, 0.77)	0.25 (−0.28, 0.78)	−0.13 (−0.95, 0.68)	0.01 (−0.52, 0.54)	PDP						
−0.16 (−0.75, 0.43)	−0.29 (−0.97, 0.38)	−0.68 (−1.61, 0.25)	−0.54 (−1.22, 0.14)	−0.55 (−1.13, 0.03)	GT					
*0.42* (*0.11, 0.73*)	0.29 (−0.16, 0.74)	−0.10 (−0.88, 0.68)	0.04 (−0.40, 0.48)	0.04 (−0.32, 0.39)	*0.58* (*0.01, 1.15*)	DBT				
0.55 (−0.06, 1.16)	0.42 (−0.19, 1.03)	−0.04 (−0.91, 0.98)	0.18 (−0.53, 0.89)	0.17 (−0.45, 0.79)	0.71 (−0.06, 1.49)	0.13 (−0.45, 0.72)	TFP			
*1.14* (*0.48, 1.80*)	*1.01* (*0.25, 1.77*)	0.62 (−0.35, 1.59)	0.76 (−0.01, 1.54)	*0.76* (*0.04, 1.48*)	*1.30* (*0.45, 2.15*)	*0.72* (*0.03, 1.41*)	0.59 (−0.10, 1.27)	ST		
0.54 (−0.02, 1.10)	0.40 (−0.19, 0.99)	0.02 (−0.89, 0.93)	0.16 (−0.51, 0.82)	0.15 (−0.40, 0.70)	*0.70* (*0.09, 1.31*)	0.12 (−0.43, 0.66)	−0.02 (−0.75, 0.72)	−0.60 (−1.43, 0.22)	MBT	
0.24 (−0.23, 0.70)	0.11 (−0.53, 0.74)	−0.28 (−1.13, 0.57)	−0.14 (−0.67, 0.39)	−0.15 (−0.69, 0.40)	0.40 (−0.23, 1.03)	−0.18 (−0.66, 0.29)	−0.32 (−1.04, 0.41)	*−0.90* (*−1.69, −0.12*)	−0.30 (−0.96, 0.36)	CBT

CBT, cognitive behaviour therapy; CTBE, community treatment by experts; DBT, dialectical behaviour therapy; GT, generic treatments; IPT, interpersonal psychotherapy; MBT, mentalisation-based therapy; Mixed, mixed therapeutic techniques; PDP, psychodynamic psychotherapy; ST, schema therapy; TAU, treatment-as-usual; TFP, transference-focused therapy.

The diagonal illustrates the different nodes that were examined in this study. Effect sizes are illustrated as SMD with 95% CIs. Data in bold and underlined are statistically significant. Comparisons between treatments should be read from left to right, and the estimate is in the cell in common between the column-defining treatment and the row-defining treatment. Negative values indicate that the row-defining intervention is less efficacious than the column-defining intervention.

Efficacy at post-test (SMD with 95% CI).

Results of the local inconsistency tests are presented in online Supplementary Figure S1a. The highest inconsistency factor was found for the loop CBT, DBT, and GT, but was not statistically significant (*p* = 0.061), indicating that direct and indirect evidence within this loop is not in conflict. In this analysis, 15% of the loops were inconsistent (3 of 20 loops; *p* value of the design-by-treatment interaction model was 0.62 and did not indicate global inconsistency in the network (χ^2^ = 9.94, df = 12)). Our comparison-adjusted funnel plot does not suggest publication bias since no asymmetry was detected (online Supplementary Figure S2a).

**Fig. 3.**
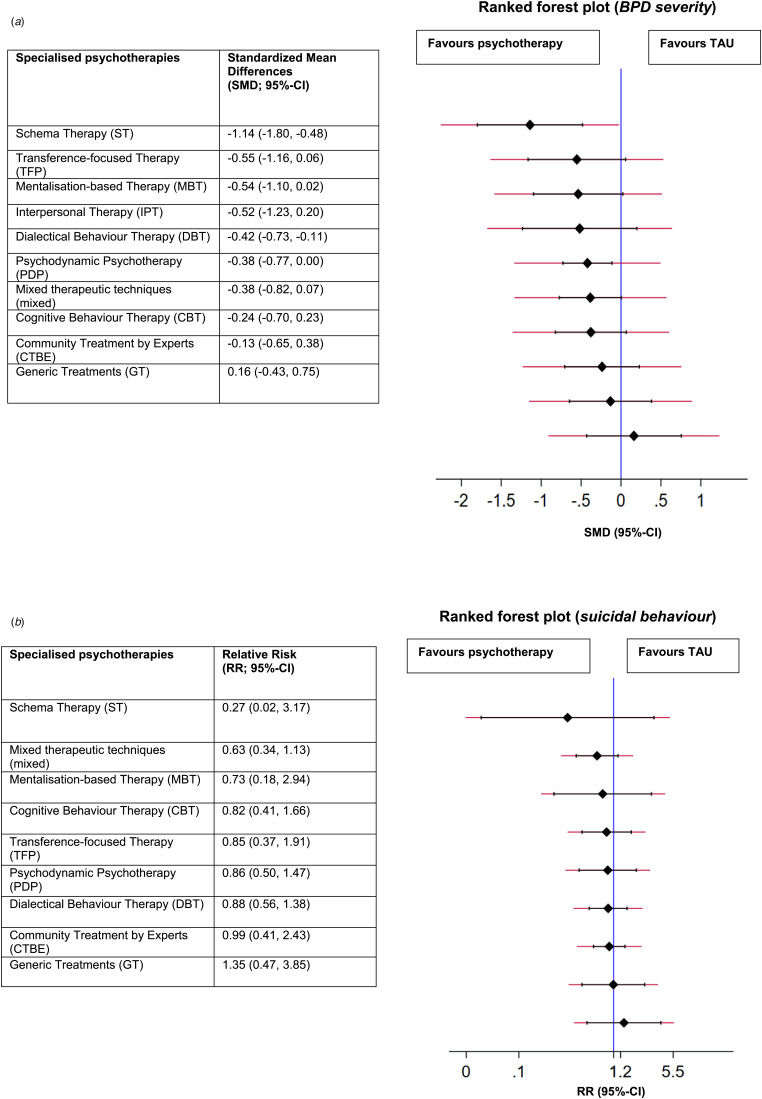
(*a*). Ranked forest plot on the efficacy of specialised psychotherapies in the treatment of BPD severity. (*b*) Ranked forest plot on the efficacy of specialised psychotherapies in the treatment of suicidal behaviour.

The results of the analyses on the ranking of psychotherapies (SUCRA) are shown in Table 4. In Fig. 3 forest plot, the treatments are ranked, with TAU as the reference group. In terms of efficacy, ST and DBT were significantly better when compared to TAU (Fig. 3a).

**Table 4.** Ranking of psychotherapies on BPD symptom severity- (left column) and suicidal behaviour (right column) by surface under the cumulative ranking curve

	Primary study outcomes			
	Overall BPD severity		Suicidal behaviour	
	Psychotherapies	Effectiveness (%)	Psychotherapies	Effectiveness (%)
1	Schema Therapy (ST)	96.8	Schema Therapy	82.2
2	Mentalisation-based therapy (MBT)	68.2	Mixed therapeutic interventions (mixed)	73.9
3	Transference-focused therapy (TFP)	67.6	Mentalisation-based therapy (MBT)	58.3
4	Interpersonal Psychotherapy (IPT)	63.2	Cognitive Behaviour Therapy (CBT)	52.5
5	Dialectical Behaviour Therapy (DBT)	58.9	Psychodynamic Psychotherapy (PDP)	49.3
6	Psychodynamic Psychotherapy (PDP)	54.0	Transference-focused therapy (TFP)	48.8
7	Mixed therapeutic interventions (mixed)	52.9	Dialectical Behaviour Therapy (DBT)	45.9
8	Cognitive Behaviour Therapy (CBT)	38.5	Community Treatment by Experts (CTBE)	37.2
9	Community Treatment by Experts (CTBE)	27.7	Treatment-As-Usual (TAU)	32.8
10	Treatment-As-Usual (TAU)	13.7	Generic Treatments (GT)	19.2
11	Generic Treatments (GT)	8.5	–	–

### Suicidal behaviour

#### Pairwise meta-analyses

The results of the pairwise meta-analyses with suicidal behaviour as the outcome variable are illustrated in online Supplementary Table S6.

#### Network plot

Of 43 RCTs, 38 studies measured suicidal behaviour. One study (Laurenssen et al., 2018) did measure suicidal behaviour, but did not report their findings in the paper. Data were not provided by the authors after contacting them, and therefore excluded from the analysis. Data from one study (Giesen-Bloo et al., 2006) was provided by one of the co-authors (AA). Studies were also excluded if they did not measure suicidal behaviour as the number of participants who engaged in suicidal behaviour, but rather as the number of events/number of days (Bellino et al., 2010; Bozzatello & Bellino, 2020; Dixon-Gordon, Chapman, & Turner, 2015; Leppanen, Hakko, Sintonen, & Lindeman, 2015; Priebe et al., 2012; 2009; Turner, 2000; Walton, Bendit, Baker, Carter, & Lewin, 2020; Visintini et al., 2020). This resulted in 29 studies (*n* = 2383). A network plot is illustrated in Fig. 2b, including a total of ten nodes (CBT (*n* = 224), MBT (*n* = 133), DBT (*n* = 561), TFP (*n* = 128), ST (*n* = 43), GT (*n* = 146), PDP (*n* = 207), mixed (*n* = 372), CTBE (*n* = 181), TAU (*n* =388)). Again, DBT *v.* TAU was the most examined comparison (*N*_comparisons_ = 6). Each node was connected to at least two other nodes within the network (except for ST). A contribution plot is provided in online Supplementary Table S5b.

#### Network meta-analysis

The main results of the NMA are presented in Table 5. No psychotherapy appeared to be significantly superior compared to TAU. Also, not any type of psychotherapy showed significant differences when compared to other psychotherapies within the network. We were unable to perform an NMA on long-term effects, because not enough studies were available (*n* = 12) (ranging from 7 weeks to 260 weeks) (Table 2). No significant inconsistency factors were found by local inconsistency tests (online Supplementary Figure S1b), and no indications for global inconsistency in the network was found (χ^2^ = 4.35, df = 9; *p* for the null hypothesis of consistency in the network: 0.88). Our comparison-adjusted funnel plot does not suggest publication bias since no asymmetry was detected (online Supplementary Figure S2b).

**Table 5.** Relative effect sizes (RRs with 95% Cs) for psychotherapies on suicidal behaviour according to network meta-analysis

TAU									
1.01 (0.41, 2.46)	CTBE								
1.60 (0.88, 2.90)	1.59 (0.57, 4.42)	Mixed							
1.17 (0.68, 2.00)	1.16 (0.48, 2.83)	0.73 (0.36, 1.50)	PDP						
0.74 (0.26, 2.13)	0.74 (0.20, 2.67)	0.46 (0.15, 1.45)	0.64 (0.25, 1.61)	GT					
1.13 (0.72, 1.77)	1.12 (0.50, 2.53)	0.71 (0.37, 1.36)	0.97 (0.62, 1.52)	1.52 (0.55, 4.21)	DBT				
1.18 (0.52, 2.67)	1.17 (0.50, 2.74)	0.74 (0.29, 1.89)	1.01 (0.48, 2.11)	1.59 (0.49, 5.17)	1.04 (0.51, 2.15)	TFP			
3.71 (0.32, 43.59)	3.69 (0.31, 43.77)	2.32 (0.19, 28.50)	3.18 (0.28, 36.42)	4.99 (0.37, 67.66)	3.28 (0.29, 37.46)	3.14 (0.31, 32.14)	ST		
1.36 (0.34, 5.47)	1.35 (0.28, 6.64)	0.85 (0.21, 3.43)	1.17 (0.33, 4.09)	1.83 (0.79, 4.27)	1.21 (0.32, 4.60)	1.15 (0.27, 5.00)	0.37 (0.02, 5.73)	MBT	
1.22 (0.60, 2.46)	1.21 (0.41, 3.61)	0.76 (0.31, 1.87)	1.04 (0.45, 2.41)	1.64 (0.49, 5.52)	1.08 (0.50, 2.31)	1.03 (0.37, 2.89)	0.33 (0.03, 4.17)	0.89 (0.20, 4.08)	CBT

CBT, cognitive behaviour therapy; CTBE, community treatment by experts; DBT, dialectical behaviour therapy; GT, generic treatments; MBT, mentalisation-based therapy; Mixed, mixed therapeutic techniques; PDP, psychodynamic psychotherapy; ST, schema therapy; TAU, treatment-as-usual; TFP, transference-focused therapy.

The diagonal illustrates the different nodes that were examined in this study. Comparisons between treatments should be read from left to right, and the estimate is in the cell in common between the column-defining treatment and the row-defining treatment. Relative risks (RRs with 95% CIs) smaller than 1 favour the column-defining treatment. To obtain RRs for comparisons in the opposite direction, reciprocals should be taken. Data underlined is statistically significant.

Efficacy at post-test (RR with 95%CI).

In Fig. 3b forest plot, the treatments are ranked, with TAU as the reference group. ST (SUCRA = 82.2%), and mixed (SUCRA = 73.9%) were ranked best (Table 4). As shown in Fig. 3b, no type of psychotherapy was significantly more beneficial in reducing suicidal behaviour when compared to TAU.

### Study drop-out

**Fig. 4.**
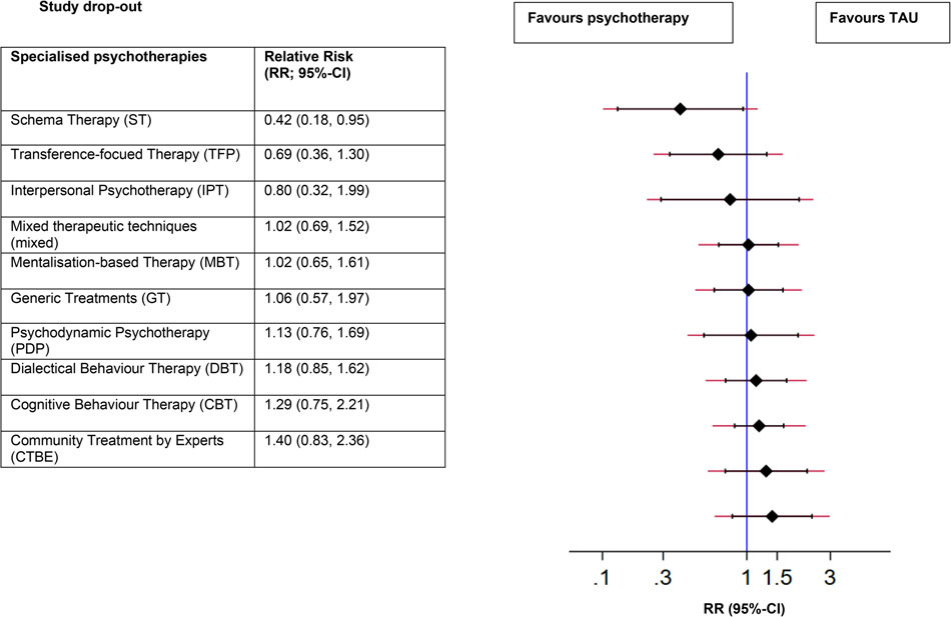
Ranked forest plot on study drop-out (for any reason) of specialised psychotherapies.

The main results of the NMA on study drop-out are presented in Table 6. TFP and ST showed significantly lower study drop-out rates compared to CTBE. Next, significantly more people dropped out during study in DBT, PDP, MBT, CBT, TAU and mixed nodes compared to ST. No other significant differences were found. In Fig. 4 forest plot, the treatments are ranked, with TAU as the reference group. ST was ranked best (SUCRA = 96.7%), followed by TFP (80.3%) (Table 7). For study drop-out, our comparison-adjusted funnel plot does not suggest publication bias since no asymmetry was detected (online Supplementary Figure S2c).

**Table 6.** Relative risks (RRs) of study drop-out for psychotherapies at post-test according to network meta-analysis

TAU										
0.72 (0.42, 1.21)	CTBE									
1.25 (0.50, 3.09)	1.74 (0.61, 4.97)	IPT								
0.98 (0.66, 1.45)	1.37 (0.78, 2.37)	0.78 (0.29, 2.11)	Mixed							
0.88 (0.59, 1.32)	1.24 (0.73, 2.10)	0.71 (0.26, 1.92)	0.91 (0.57, 1.45)	PDP						
0.95 (0.51, 1.76)	1.32 (0.67, 2.59)	0.76 (0.25, 2.28)	0.97 (0.52, 1.82)	1.07 (0.57, 2.01)	GT					
0.85 (0.62, 1.17)	1.19 (0.73, 1.93)	0.68 (0.26, 1.79)	0.87 (0.60, 1.26)	0.96 (0.68, 1.35)	0.90 (0.49, 1.65)	DBT				
1.46 (0.77, 2.76)	*2.03* (*1.10, 3.76*)	1.17 (0.39, 3.55)	1.49 (0.76, 2.93)	1.65 (0.87, 3.11)	1.54 (0.69, 3.45)	1.71 (0.94, 3.13)	TFP			
*2.40* (*1.05, 5.48*)	*3.35* (*1.43, 7.86*)	1.93 (0.56, 6.57)	*2.45* (*1.03, 5.86*)	*2.71* (*1.17, 6.30*)	2.53 (0.95, 6.77)	*2.82* (*1.25, 6.38*)	1.65 (0.84, 3.22)	ST		
0.98 (0.62, 1.54)	1.36 (0.84, 2.22)	0.78 (0.28, 2.16)	1.00 (0.60, 1.67)	1.10 (0.71, 1.71)	1.03 (0.60, 1.78)	1.15 (0.74, 1.78)	0.67 (0.35, 1.30)	*0.41 (0.17, 0.97)*	MBT	
0.77 (0.45, 1.33)	1.08 (0.57, 2.07)	0.62 (0.22, 1.79)	0.79 (0.48, 1.30)	0.88 (0.49, 1.57)	0.82 (0.46, 1.47)	0.91 (0.54, 1.53)	0.53 (0.25, 1.14)	*0.32 (0.13, 0.83)*	0.79 (0.44, 1.42)	CBT

CBT, cognitive behaviour therapy; CTBE, community treatment by experts; DBT, dialectical behaviour therapy; GT, generic treatments; IPT, interpersonal psychotherapy; MBT, mentalisation-based therapy; Mixed, mixed therapeutic techniques; PDP, psychodynamic psychotherapy; ST, schema therapy; TAU, treatment-as-usual; TFP, transference-focused therapy.

The diagonal illustrates the different nodes that were examined in this study. Effect sizes are illustrated as RRs with 95% CIs. Data underlined is statistically significant. Comparisons between treatments should be read from left to right, and the estimate is in the cell in common between the column-defining treatment and the row-defining treatment. RRs with 95% CIs larger than 1 favour the row-defining treatment. To obtain RRs for comparisons in the opposite direction, reciprocals should be taken.

Study drop-out at post-test (RR with 95% CI).

**Table 7.** Ranking of psychotherapies on study drop-out by surface under the cumulative ranking curve

	Psychotherapies	Study drop-out (%)
1	Schema Therapy (ST)	96.7
2	Transference-focused Therapy (TFP)	80.3
3	Interpersonal Psychotherapy (IPT)	64.6
4	Treatment-As-Usual (TAU)	54.4
5	Mixed therapeutic interventions (mixed)	50.8
6	Mentalisation-based Therapy (MBT)	50.5
7	Generic Treatments (GT)	45.2
8	Psychodynamic Psychotherapy (PDP)	37.0
9	Dialectical Behaviour Therapy (DBT)	30.9
10	Cognitive Behaviour Therapy (CBT)	23.7
11	Community Treatment by Experts (CTBE)	16.0

### Sensitivity analyses

Firstly, we performed a sensitivity analysis with only studies investigating a ‘full DBT’ intervention. The findings were comparable to the main analyses, with overlap in the direction of effect estimates, and similar 95% CIs (online Supplementary Tables S7 & S11). Secondly, we limited the analysis to studies purely measuring suicidal behaviour (death by suicide and suicide attempts). The network was less populated, including 23 studies, reporting wider, but overlapping 95% CIs, meaning that these findings were comparable to the main results, whereby no psychotherapy was significantly better compared to the others (online Supplementary Tables S8 & S12). Next, because each included study contained a considerable RoB, no trial was assessed as high methodological quality. We therefore could not perform a sensitivity analysis on studies with low RoB. Fourth, we performed a sensitivity analysis by excluding 11 studies that did not measure overall BPD severity (online Supplementary Tables S9 & S11). Although the outcomes were not identical in terms of statistical significance and (direction of) effect estimates, and reported wider 95% CIs, the majority of findings still overlapped. Finally, we performed a sensitivity analysis by only including studies using a combined format (individual + group). The network included a total of 20 studies. Due to a lack of studies, two nodes were excluded from the sensitivity analysis (ST, and IPT). Results showed that, although the network was less populated, there was still overlap in the direction of the effect estimates and reported 95% CIs, making these findings comparable to the main analyses (online Supplementary Tables S10 & S13).

## Discussion

This is the first NMA to directly compare the effectiveness of different types of psychotherapies in the treatment of people with BPD. Study drop-out was included as a secondary outcome. We found that DBT, MBT and ST were significantly more effective compared to TAU and GT. These findings remained statistically significant after performing sensitivity analyses only including ‘full’ DBT-interventions, and therapies using combined formats (individual + group). Despite that the DBT effect size was smaller than other treatments, this intervention does have a more robust evidence-base given that half of all studies in this NMA (19 out of 43 RCTs) investigated the efficacy of DBT. Next, ST appeared to be more effective in treating borderline severity in adults diagnosed with BPD than several other active treatments, including CBT, DBT, and PDP, however, these comparisons were only based on three trials, so this should be considered with caution. Between the remaining therapies included in our NMA, no significant differences were found. Possible explanations might be a lack of robust evidence-base and statistical power, and smaller number of included trials.

With regards to suicidal behaviour, no psychotherapy appeared to be significantly superior compared to TAU or to each other. Several treatments (see Table 5) do seem to be promising (RR around 0.5), meaning that these psychotherapies tend to reduce suicidal behaviour with almost 50% within this participant group by the end of treatment. Suicidal behaviour was investigated in only a small number of trials, and in combination with low N in most study arms and nodes, it is much more difficult to successfully detect any significant effects. ST and TFP reported lower drop-out rate compared to other treatments such as CTBE. However, the ST and TFP-nodes included only a few studies, and that is why its results should be interpreted carefully.

Our NMA is in partial agreement with previous reviews, suggesting that DBT is effective to treat BPD compared to TAU (Cristea et al., 2017; Storebo et al., 2020). However, in contrast to Cristea et al., no beneficial effects for PDP in comparison with TAU were found. This could be explained by the fact that Cristea et al., grouped all (supportive and explorative) psychodynamic approaches together (MBT, TFP, PDP), instead of breaking them down into their own category, resulting in a smaller number of trials for each group. Compared to the review by (Oud et al., 2018), we took a broader approach and included more psychotherapies. Our results partly overlap with those of Storebo et al. (2020), as we found no significant differences between the majority of included BPD-tailored therapies, nevertheless, therapies such as DBT did provide solid evidence on its effectiveness compared to less intensive or specialised therapies such as GT, CTBE, or TAU. Further, the meta-analysis by (Stoffers-Winterling et al., 2022), examined the effectiveness of stand-alone and add-on therapies for BPD. Although they do conclude that the impacts for some therapies are promising, they were not able to make more than two comparisons between similar treatments. For example, their study found no significant effects for DBT *v.* TAU on BPD severity, whilst in our study, the effect estimate for DBT appeared to be very stable and significant compared to TAU. They also found a superior effect for DBT and MBT *v.* TAU on self-harm and suicide-related outcomes, but this was only based on a pairwise comparison from two or three trials. Also, they were unable to include other specialised treatments (ST, TFP) due to a lack of trials. Our NMA approach made it possible exploit the data more efficiently, and to compare different types of specialised psychological treatments in such a way, no prior meta-analysis was able to do so before.

### Limitations and strengths

This study has several limitations. First, we detected variability across included studies in terms of control condition, treatment dose, formats, measurements and reporting and selection of study outcomes, and methodological quality. Some therapies were reported in smaller number of trials. Also, there are differences in treatment dose (e.g. therapy duration and number of sessions) and used formats between studies (group + individual or individual/group alone). Control conditions across RCTs tend to differ considerably. Trials often chose different study outcomes, or used different scales or interviews to measure a similar concept (e.g. overall BPD severity). Therefore, it would be helpful if future studies would select similar measurements based on the recommendations of the International Consortium for Health Outcomes Measurement (Prevolnik Rupel et al., 2021), such as the Zanarini Rating Scale for BPD (ZAN-BPD), the Borderline Symptom List (BSL), or the BPD Severity Index (BPDSI) (Zanarini et al., 2010). Next to that, future studies should also pay more attention onto how they report their findings. For example, some trials did use valid instruments to measure their outcomes, however, we could still not meta-analyse them because results were not adequately reported, or were not extractable from the included studies. Second, in this study we measured study drop-out for any reason, and not treatment drop-out, because the latter one was often defined differently across trials, making it difficult to statistically pool and interpret results. To gain more insight into the actual treatment acceptability, future studies should measure treatment drop-out more homogeneously, for example by using similar definitions. A recent meta-analysis by Arntz et al. (2022) investigated dropout rates from psychological treatments for BPD. Based on their findings, they also recommend future studies to better distinguish between different types of dropout (treatment dropout *v.* study dropout), by defining them more clearly according to guidelines [e.g. CONSORT guidelines (Schulz, Altman, Moher, & Group, 2010)]. Third, we might have introduced heterogeneity for some nodes, such as for TAU. Despite these limitations, our sensitivity analyses produced quite similar results to the main analyses, indicating that our conclusions are robust. Fourth, we were able to include 43 studies, but not all studies could be included in the statistical analyses. Only a minimal number of studies were available for direct comparisons. Fifth, insufficient numbers of studies were available on long-term effects. Most studies were unclear in reporting their treatment drop-out rate, defined it inconsistently, or provided insufficient information to infer it, which made it difficult to statistically pool the results. Finally, some studies measured multiple outcomes (i.e. self-harm and suicide attempts) within a similar scale, not reporting the results separately.

### Implications for research and practice

We also have a few recommendations. First, because we did not find any significant differences between specialised treatments, one might argue that future studies should try to replicate the effects of already existing treatments preferably in head-to-head trials, instead of developing new treatments or ‘modified/simplified’ interventions (Oud et al., 2018). However, head-to-head trials do not generally suggest superiority of any of the available specialised treatments for BPD relative to one another. Building up an evidence-base of RCTs could therefore be helpful to examine whether there are indeed no differences between therapies. However, it is the question if future RCTs would be able to provide a definitive answer. On the other hand, specialised treatments are warranted for most people with BPD (Barnicot et al., 2012), and one may argue whether it is a bad thing when people can choose from an array of empirically support treatment options available. In general, people benefit more from their treatment if they receive a therapy based on their own preference (Mergl et al., 2011; Swift, Callahan, & Vollmer, 2011). Also, they are less likely to drop-out, and specialised therapies are more cost saving when compared to TAU (Brettschneider, Riedel-Heller, & König, 2014). These costs can be reduced further if studies support the development of tailored specialised therapies for specific BPD-profiles (Oud et al., 2018). Second, although large RRs of 0.5 between studies were found, no psychotherapy was significantly superior compared to others in reducing suicidal behaviour. Previous studies have suggested that especially treatments directly targeting suicidal behaviour are more effective compared to therapies using indirect approaches (van Meerwijk et al., 2016). In our study, DBT is one of the few therapies that directly targets suicidal behaviour, however, its effects were not significantly better compared to other therapies. One explanation could be that DBT was compared to other ‘active’ nodes, including trials that are also known for their primary focus on suicidal behaviour such as CAMS (Andreasson et al., 2016). Third, the evidence-base for some therapies is still limited, given the small number of studies and sample sizes. On the other hand, more large-scale RCTs are currently underway. For example, Arntz et al. (2022) published an RCT (*n* = 495), and reported similar effect sizes for ST compared to TAU on BPD-severity as the present NMA. Fourth, we noticed that not all RCTs including participants with BPD actually measured overall BPD severity. We would therefore advise future studies to include validated assessments. Fifth, large-scale RCTs using follow-up measurements are highly warranted to investigate long-term effects. Sixth, more research onto the working mechanisms and mediators of psychotherapies is very important. However, it is difficult to show how a therapy exactly works, and it is therefore still unknown whether therapies work through common or specific factors, or both (Cuijpers, Reijnders, & Huibers, 2019). Finally, given the heterogeneity and complexity of BPD and the range of specialised psychotherapies available, persons with a certain combination of BPD symptoms might benefit more or less from different types of psychotherapy. By identifying these patient characteristics, it may allow mental health services to provide more tailored and individualised treatments, thereby optimising the quality of care for people with BPD. In order to move towards a more personalised approach, it might be worthwhile to undertake an individual participant data meta-analysis (IPDMA) in the near future (Storebø et al., 2020).

## Conclusion

In conclusion, the evidence from this study is not strong enough to provide a clear answer to the question whether one single therapy is significantly more efficacious in treating BPD symptoms compared to others, and if so, which one. Although a few therapies showed a significant improvement in BPD symptoms compared to other therapies, these findings were based on very few trials and should therefore be interpreted with caution. With regards to suicidal behaviour, some treatments almost halved the risk of attempted suicide and committed suicide (combined rate), reporting RRs around 0.5, but these results were not significant compared to other therapies or TAU. We therefore suggest that future studies should investigate the efficacy of these existing treatments more extensively in high-quality RCTs, preferably in head-to-head trials using direct evidence. We advise future studies to conduct an IPDMA. This might help to shed light on potential moderators and predictors, providing information on who might benefit more or less from different types of psychotherapies (Storebø et al., 2020).

## Data Availability

All records and data of this study are saved in a separate database. If researchers are interested in the data, they can contact us by using the contact information of the corresponding author (KS).

## Supplementary material

For supplementary material accompanying this paper visit https://doi.org/10.1017/S0033291723000685. click here to view supplementary material
