# Supplementary material for: Which psychotherapy is most effective and acceptable in the treatment of adults with a (sub)clinical borderline personality disorder? A systematic review and network meta-analysis
Source: Psychol Med. 2023 May 19;53(8):3261–80. doi: 10.1017/S0033291723000685 (PMC10277776; doi:10.1017/S0033291723000685)

**Supplement**

Which psychotherapy is most effective and acceptable in the treatment of adults with a (sub)clinical borderline personality disorder?

A systematic review and network meta-analysis

Kim Setkowski et al.

**Supplementary Table 1.**

**Complete search string (PubMed)**

**# Personality disorders (borderline symptoms)**

“borderline client*”[tiab] OR “borderline patient*”[tiab] OR “borderline person*”[tiab] OR "Borderline Personality Disorder"[Mesh] OR “borderline state*”[tiab] OR “borderline symptom*”[tiab] OR “personality dis*”[tiab] OR "Personality Disorders"[Mesh]

**# Psychotherapy**

“acceptance and commitment therap*”[tiab] OR aromatherap*[tiab] OR “art therap*”[tiab] OR “behavior intervention*”[tiab] OR “behavior therap*”[tiab] OR “behavior treatment*”[tiab] OR “behavioral intervention*”[tiab] OR “behavioral therap*”[tiab] OR “behavioral treatment*”[tiab] OR “behaviour intervention*”[tiab] OR “behaviour therap*”[tiab] OR “behaviour treatment*”[tiab] OR “behavioural intervention*”[tiab] OR “behavioural therap*”[tiab] OR “behavioural treatment*”[tiab] OR bibliotherap*[tiab] OR CAMS[tiab] OR CBT[tiab] OR “cognitive psychotherap*”[tiab] OR “cognitive therap*”[tiab] OR “collaborative assessment*”[tiab] OR “color therap*”[tiab] OR “crisis intervention*”[tiab] OR “dance therap*”[tiab] OR (DBT[tiab] AND dialectical*[tiab]) OR “desensitisation reprocessing”[tiab] OR “desensitization reprocessing”[tiab] OR “dialectical behavio*”[tiab] OR EMDR[tiab] OR “emotion-focused therap*”[tiab] OR “exposure therap*”[tiab] OR (“future oriented”[tiab] AND training[tiab]) OR “gestalt therap*”[tiab] OR grouptherap*[tiab] OR “group therap*”[tiab] OR hypnos*[tiab] OR imagery[tiab] OR “implosive therap*”[tiab] OR “interpersonal therap*”[tiab] OR “mentalisation-based therap*”[tiab] OR “mentalization-based therap*”[tiab] OR (“mentalisation-based”[tiab] AND training[tiab]) OR (“mentalization-based”[tiab] AND training[tiab]) OR “milieu therap*”[tiab] OR mindfulness[tiab] OR “music therap*”[tiab] OR “narrative therap*”[tiab] OR “play therap*”[tiab] OR “problem-solving therap*”[tiab] OR “psychoanalytic therap*”[tiab] OR “psychological desensitization*”[tiab] OR “psychological feedback”[tiab] OR “psychological intervention*”[tiab] OR “psychological therap*”[tiab] OR “psychological treatment*”[tiab] OR psychotherap*[tiab] OR "Psychotherapy"[Mesh] OR “reality therap*”[tiab] OR “sensory feedback”[tiab] OR “socioenvironmental therap*”[tiab] OR STEPPS[tiab] OR (systems[tiab] AND training[tiab] AND emotional[tiab] AND predictability[tiab] AND “problem solving*”[tiab])

**# Studytype**“clinical trial*”[tiab] OR “Clinical Trial”[pt] OR "Clinical Trials as Topic"[Mesh] OR “Controlled Clinical Trial”[pt] OR “controlled trial*”[tiab] OR “Double-blind method”[Mesh] OR feasib* [tiab] OR "Feasibility Studies"[Mesh] OR random [tw] OR “Random allocation” [Mesh] OR randomised[tw] OR randomising[tw] OR randomized[tw] OR “Randomized Controlled Trial”[pt] OR “Randomized Controlled Trials as Topic”[Mesh] OR randomizing [tw] OR randomly[tw] OR RCT[tiab] OR ((singl*[tw] OR doubl*[tw] OR trebl*[tw] OR tripl*[tw]) AND (mask*[tw] OR blind*[tw] OR dumm*[tw])) OR “Single-blind method”[Mesh] OR utilit*[tiab]

**Complete search string (Embase)**

**# Personality disorders (borderline symptoms)**

‘borderline client*’:ab,ti,kw OR ‘borderline patient*’:ab,ti,kw OR ‘borderline person*’:ab,ti,kw OR ‘borderline state*’:ab,ti,kw OR 'borderline state'/exp OR ‘borderline symptom*’:ab,ti,kw OR ‘personality dis*’:ab,ti,kw OR 'personality disorder'/exp

**# Psychotherapy**

‘acceptance and commitment therap*’:ab,ti,kw OR aromatherap*:ab,ti,kw OR ‘art therap*’:ab,ti,kw OR ‘behavior intervention*’:ab,ti,kw OR ‘behavior therap*’:ab,ti,kw OR ‘behavior treatment*’:ab,ti,kw OR ‘behavioral therap*’:ab,ti,kw OR ‘behavioral intervention*’:ab,ti,kw OR ‘behavioral treatment*’:ab,ti,kw OR ‘behaviour intervention*’:ab,ti,kw OR ‘behaviour therap*’:ab,ti,kw OR ‘behaviour treatment*’:ab,ti,kw OR ‘behavioural intervention*’:ab,ti,kw OR ‘behavioural therap*’:ab,ti,kw OR ‘behavioural treatment*’:ab,ti,kw OR bibliotherap*:ab,ti,kw OR CAMS:ab,ti,kw OR CBT:ab,ti,kw OR ‘cognitive psychotherap*’:ab,ti,kw OR ‘cognitive therap*’:ab,ti,kw OR ‘collaborative assessment*’:ab,ti,kw OR ‘color therap*’:ab,ti,kw OR ‘crisis intervention*’:ab,ti,kw OR ‘dance therap*’:ab,ti,kw OR (DBT NEAR/3 dialectical*):ab,ti,kw OR ‘desensitisation reprocessing’:ab,ti,kw OR ‘desensitization reprocessing’:ab,ti,kw OR ‘dialectical behavio*’:ab,ti,kw OR EMDR:ab,ti,kw OR ‘emotion-focused therap*’:ab,ti,kw OR ‘exposure therap*’:ab,ti,kw OR (‘future oriented’ NEAR/3 training):ab,ti,kw OR ‘gestalt therap*’:ab,ti,kw OR ‘group therap*’:ab,ti,kw OR grouptherap*:ab,ti,kw OR hypnos*:ab,ti,kw OR imagery:ab,ti,kw OR ‘implosive therap*’:ab,ti,kw OR ‘interpersonal therap*’:ab,ti,kw OR (‘mentalisation-based’ NEAR/3 training):ab,ti,kw OR (‘mentalization-based’ NEAR/3 training):ab,ti,kw OR ‘mentalisation-based therap*’:ab,ti,kw OR ‘mentalization-based therap*’:ab,ti,kw OR ‘milieu therap*’:ab,ti,kw OR mindfulness:ab,ti,kw OR ‘music therap*’:ab,ti,kw OR ‘narrative therap*’:ab,ti,kw OR ‘play therap*’:ab,ti,kw OR ‘problem-solving therap*’:ab,ti,kw OR ‘psychoanalytic therap*’:ab,ti,kw OR ‘psychological desensitization*’:ab,ti,kw OR ‘psychological feedback’:ab,ti,kw OR ‘psychological intervention*’:ab,ti,kw OR ‘psychological therap*’:ab,ti,kw OR ‘psychological treatment*’:ab,ti,kw OR psychotherap*:ab,ti,kw OR 'psychotherapy'/exp OR ‘reality therap*’:ab,ti,kw OR ‘sensory feedback’:ab,ti,kw OR ‘socioenvironmental therap*’:ab,ti,kw OR STEPPS:ab,ti,kw OR (systems NEAR/1 training NEAR/2 emotional NEAR/1 predictability NEAR/2 ‘problem solving*’):ab,ti,kw

**# Studytype**‘controlled trial*':ab,ti,kw OR ‘clinical trial*’:ab,ti,kw OR 'clinical trial'/de OR 'clinical trial'/lnk OR 'controlled clinical trial'/de OR 'controlled clinical trial'/exp OR 'controlled study'/de OR 'double blind procedure'/de OR feasib*:ab,ti,kw OR 'feasibility study'/de OR random:ab,ti,kw OR randomised:ab,ti,kw OR randomising:ab,ti,kw OR 'randomization'/exp OR 'randomized controlled trial'/exp OR randomized:ab,ti,kw OR randomizing:ab,ti,kw OR randomly:ab,ti,kw OR RCT*:ab,ti,kw OR 'single blind procedure'/exp OR ((singl* OR doubl* OR trebl* OR tripl*) NEAR/3 (mask* OR blind* OR dumm*)):ab,ti,kw OR utilit*:ab,ti,kw

**Complete search string (PsycINFO)**

**# Personality disorders (borderline symptoms)**

DE "Antisocial Personality Disorder" OR DE "Avoidant Personality Disorder" OR KW(borderline) OR “borderline client*” OR “borderline client*” OR “borderline patient*” OR “borderline patient*” OR “borderline person*” OR “borderline person*” OR DE "Borderline Personality Disorder" OR “borderline state*”) OR “borderline state*”) OR DE "Borderline States" OR “borderline symptom*” OR “borderline symptom*” OR DE "Dependent Personality Disorder" OR DE "Histrionic Personality Disorder" OR DE "Narcissistic Personality Disorder" OR DE "Obsessive Compulsive Personality Disorder" OR DE "Paranoid Personality Disorder" OR DE "Passive Aggressive Personality Disorder" OR AB(“personality dis*” OR TI(“personality dis*” OR DE "Personality Disorders" OR DE "Sadomasochistic Personality" OR DE "Schizoid Personality Disorder" OR DE "Schizotypal Personality Disorder"

**# Psychotherapy**
“acceptance and commitment therap*” OR “acceptance and commitment therap*” OR DE "Acceptance and Commitment Therapy" OR DE "Adlerian Psychotherapy" OR DE "Adolescent Psychotherapy" OR DE "Affirmative Therapy" OR DE "Analytical Psychotherapy" OR aromatherap* OR aromatherap* OR aromatherap* OR “art therap*” OR “art therap*” OR DE "Autogenic Training" OR “behavior intervention*” OR “behavior intervention*” OR “behavior therap*” OR “behavior therap*” OR DE "Behavior Therapy" OR “behavior treatment*” OR “behavior treatment*” OR “behavioral intervention*” OR “behavioral intervention*” OR “behavioral therap*” OR “behavioral therap*” OR “behavioral treatment*” OR “behavioral treatment*” OR “behaviour intervention*” OR “behaviour intervention*” OR “behaviour therap*” OR “behaviour therap*” OR “behaviour treatment*” OR “behaviour treatment*” OR “behavioural intervention*” OR “behavioural intervention*” OR “behavioural therap*” OR “behavioural therap*” OR “behavioural treatment*” OR “behavioural treatment*” OR bibliotherap* OR bibliotherap* OR bibliotherap* OR DE "Brief Psychotherapy" OR DE "Brief Relational Therapy" OR “CAMS” OR “CAMS” OR “CAMS” OR CBT OR CBT OR CBT OR DE "Child Psychotherapy" OR DE "Client Centered Therapy" OR DE "Cognitive Behavior Therapy" OR “cognitive psychotherap*” OR “cognitive psychotherap*” OR DE "Cognitive Restructuring" OR “cognitive therap*” OR “cognitive therap*” OR DE "Cognitive Therapy" OR “collaborative assessment*” OR “collaborative assessment*” OR “color therap*” OR “color therap*” OR DE "Conversion Therapy" OR DE "Couples Therapy" OR “crisis intervention*” OR “crisis intervention*” OR “dance therap*” OR “dance therap*” OR (DBT W3 dialectical*) OR (DBT W3 dialectical*) OR “desensitisation reprocessing” OR “desensitisation reprocessing” OR “desensitization reprocessing” OR “desensitization reprocessing” OR “dialectical behavio*” OR “dialectical behavio*” OR DE "Dialectical Behavior Therapy" OR DE "Eclectic Psychotherapy" OR “EMDR” OR “EMDR” OR “EMDR” OR “emotion-focused therap*” OR “emotion-focused therap*” OR DE "Emotion Focused Therapy" OR DE "Existential Therapy" OR DE "Experiential Psychotherapy" OR “exposure therap*” OR “exposure therap*” OR DE "Exposure Therapy" OR DE "Expressive Psychotherapy" OR DE "Eye Movement Desensitization Therapy" OR DE "Feminist Therapy" OR (“future oriented” W3 training) OR (“future oriented” W3 training) OR “gestalt therap*” OR “gestalt therap*” OR DE "Gestalt Therapy" OR DE "Group Psychotherapy" OR grouptherap* OR grouptherap* OR grouptherap* OR “group therap*” OR “group therap*” OR DE "Guided Imagery" OR DE "Humanistic Psychotherapy" OR hypnos* OR hypnos* OR hypnos* OR DE "Hypnotherapy" OR DE "Imaginal Exposure" OR imagery OR imagery OR imagery OR “implosive therap*” OR “implosive therap*” OR DE "Implosive Therapy" OR DE "In Vivo Exposure" OR DE "Individual Psychotherapy" OR DE "Insight Therapy" OR DE "Integrative Psychotherapy" OR DE "Interpersonal Psychotherapy" OR “interpersonal therap*” OR “interpersonal therap*” OR DE "Logotherapy" OR “mentalisation-based therap*” OR “mentalisation-based therap*” OR (“mentalisation-based” W3 training) OR (“mentalisation-based” W3 training) OR “mentalization-based therap*”) OR “mentalization-based therap*”) OR (“mentalization-based” W3 training) OR (“mentalization-based” W3 training) OR “milieu therap*” OR “milieu therap*” OR mindfulness OR mindfulness OR mindfulness OR DE "Mindfulness" OR DE "Mindfulness-Based Interventions" OR DE "Multisystemic Therapy" OR “music therap*” OR “music therap*” OR “narrative therap*” OR “narrative therap*” OR DE "Narrative Therapy" OR DE "Network Therapy" OR DE "Persuasion Therapy" OR “play therap*” OR “play therap*” OR DE "Play Therapy" OR “problem-solving therap*” OR “problem-solving therap*” OR DE "Prolonged Exposure Therapy" OR “psychoanalytic therap*” OR “psychoanalytic therap*” OR DE "Psychodrama" OR DE "Psychodynamic Psychotherapy" OR “psychological desensitization*” OR “psychological desensitization*” OR “psychological feedback” OR “psychological feedback” OR “psychological intervention*” OR “psychological intervention*” OR “psychological therap*” OR “psychological therap*” OR “psychological treatment*” OR “psychological treatment*” OR AB(psychotherap* OR KW(psychotherap* OR TI(psychotherap* OR DE "Psychotherapeutic Counseling" OR DE "Psychotherapy" OR DE "Rational Emotive Behavior Therapy" OR “reality therap*” OR “reality therap*” OR DE "Reality Therapy" OR DE "Schema Therapy" OR “sensory feedback” OR “sensory feedback” OR “socioenvironmental therap*” OR “socioenvironmental therap*” OR DE "Solution Focused Therapy" OR “STEPPS” OR “STEPPS” OR “STEPPS”) OR DE "Strategic Therapy" OR DE "Supportive Psychotherapy" OR DE "Systematic Desensitization Therapy" OR (“systems training” W1 “emotional predictability” W1 “problem solving*”) OR (“systems training” W1 “emotional predictability” W1 “problem solving*”) OR DE "Transactional Analysis" OR DE "Virtual Reality Exposure Therapy"

**# Studytype**DE "Clinical Trials" OR DE "Randomized Clinical Trials" OR DE "Randomized Controlled Trials" OR AB(random OR randomly OR randomised OR randomized OR randomising OR randomizing OR ((singl* OR doubl* OR trebl* OR tripl*) W3 (mask* OR blind* OR dumm*)) OR RCT* OR feasib* OR “clinical trial*” OR “controlled trial*” OR utilit*) OR KW(random OR randomly OR randomised OR randomized OR randomising OR randomizing OR ((singl* OR doubl* OR trebl* OR tripl*) W3 (mask* OR blind* OR dumm*)) OR RCT* OR feasib* OR “clinical trial*” OR “controlled trial*” OR utilit*) OR TI(random OR randomly OR randomised OR randomized OR randomising OR randomizing OR ((singl* OR doubl* OR trebl* OR tripl*) W3 (mask* OR blind* OR dumm*)) OR RCT* OR feasib* OR “clinical trial*” OR “controlled trial*” OR utilit*) OR DE "Random Sampling"

**Complete search string (Web of Science)**

**# Personality disorders (borderline symptoms)**TS=(“borderline client*” OR “borderline patient*” OR “borderline person*” OR “borderline state*” OR “borderline symptom*” OR “personality dis*”)

**# Psychotherapy**TS=( “acceptance and commitment therap*” OR aromatherap* OR “art therap*” OR “behavior intervention*” OR “behavior therap*” OR “behavior treatment*” OR “behavioral intervention*” OR “behavioral therap*” OR “behavioral treatment*” OR “behaviour intervention*” OR “behaviour therap*” OR “behaviour treatment*” OR “behavioural intervention*” OR “behavioural therap*” OR “behavioural treatment*” OR bibliotherap* OR “CAMS” OR CBT OR “cognitive psychotherap*” OR “cognitive therap*” OR “collaborative assessment*” OR “color therap*” OR “crisis intervention*” OR “dance therap*” OR (DBT NEAR/3 dialectical*) OR “desensitisation reprocessing” OR “desensitization reprocessing” OR “dialectical behavio*” OR “EMDR” OR “emotion-focused therap*” OR “exposure therap*” OR (“future oriented” NEAR/3 training) OR “gestalt therap*” OR “group therap*” OR grouptherap* OR hypnos* OR imagery OR “implosive therap*” OR “interpersonal therap*” OR mindfulness OR (“mentalisation-based” NEAR/3 training) OR (“mentalization-based” NEAR/3 training) OR “mentalisation-based therap*” OR “mentalization-based therap*” OR “milieu therap*” OR “music therap*” OR “narrative therap*” OR “play therap*” OR “problem-solving therap*” OR “psychoanalytic therap*” OR “psychological desensitization*” OR “psychological feedback” OR “psychological intervention*” OR “psychological therap*” OR “psychological treatment*” OR psychotherap* OR “reality therap*” OR “sensory feedback” OR “socioenvironmental therap*” OR “STEPPS” OR (“systems training” NEAR/1 “emotional predictability” NEAR/1 “problem solving*”))

**# Studytype**TS=(“clinical trial*” OR “controlled trial*” OR feasib* OR random OR randomised OR randomising OR randomized OR randomizing OR randomly OR RCT* OR ((singl* OR doubl* OR trebl* OR tripl*) NEAR/3 (mask* OR blind* OR dumm*)) OR utilit*)

**Complete search string (Scopus)**

**# Personality disorders (borderline symptoms)**TITLE-ABS-KEY(“borderline client*” OR “borderline patient*” OR “borderline person*” OR “borderline state*” OR “borderline symptom*” OR “personality dis*”)

**# Psychotherapy**

TITLE-ABS-KEY(“acceptance and commitment therap*” OR “art therap*” OR aromatherap* OR “behavior intervention*” OR “behavior therap*” OR “behavior treatment*” OR “behavioral intervention*” OR “behavioral therap*” OR “behavioral treatment*” OR “behaviour intervention*” OR “behaviour therap*” OR “behaviour treatment*” OR “behavioural intervention*” OR “behavioural therap*” OR “behavioural treatment*” OR bibliotherap* OR “CAMS” OR CBT OR “cognitive psychotherap*” OR “cognitive therap*” OR “collaborative assessment*” OR “color therap*” OR “crisis intervention*” OR “dance therap*” OR (DBT W/3 dialectical*) OR “desensitisation reprocessing” OR “desensitization reprocessing” OR “dialectical behavio*” OR “EMDR” OR “emotion-focused therap*” OR “exposure therap*” OR (“future oriented” W/3 training) OR “gestalt therap*” OR “group therap*” OR grouptherap* OR hypnos* OR imagery OR “implosive therap*” OR “interpersonal therap*” OR (“mentalisation-based” W/3 training) OR (“mentalization-based” W/3 training) OR “mentalisation-based therap*” OR “mentalization-based therap*” OR “milieu therap*” OR mindfulness OR “music therap*” OR “narrative therap*” OR “play therap*” OR “problem-solving therap*” OR “psychoanalytic therap*” OR “psychological desensitization*” OR “psychological feedback” OR “psychological intervention*” OR “psychological therap*” OR “psychological treatment*” OR psychotherap* OR “reality therap*” OR “sensory feedback” OR “socioenvironmental therap*” OR “STEPPS” OR (“systems training” W/1 “emotional predictability” W/1 “problem solving*”))

**# Studytype**TITLE-ABS-KEY(“clinical trial*” OR “controlled trial*” OR feasib* OR random OR randomised OR randomising OR randomized OR randomizing OR randomly OR RCT* OR ((singl* OR doubl* OR trebl* OR tripl*) W/3 (mask* OR blind* OR dumm*)) OR utilit*)

**Complete search string (Cochrane)**

**# Personality disorders (borderline symptoms)**(“borderline client*” OR “borderline patient*” OR “borderline person*” OR “borderline state*” OR “borderline symptom*” OR “personality disorder*”):ab,ti,kw

**# Psychotherapy**(“acceptance and commitment therapy” OR aromatherap* OR “art therapy” OR “behavior intervention*” OR “behavior therapy” OR “behavior treatment*” OR “behavioral intervention*” OR “behavioral therapy” OR “behavioral treatment*” OR “behaviour intervention*” OR “behaviour therapy” OR “behaviour treatment*” OR “behavioural intervention*” OR “behavioural therapy” OR “behavioural treatment*” OR bibliotherap* OR “CAMS” OR CBT OR “cognitive psychotherapy” OR “cognitive therapy” OR “collaborative assessment*” OR “color therapy” OR “crisis intervention*” OR “dance therapy” OR (DBT AND dialectical*) OR “desensitisation reprocessing” OR “desensitization reprocessing” OR “dialectical behavioral” OR “EMDR” OR “emotion-focused therapy” OR “exposure therapy” OR (“future oriented” AND training) OR “gestalt therapy” OR “group therapy” OR grouptherapy OR hypnose OR imagery OR “implosive therapy” OR “interpersonal therapy” OR (“mentalisation-based” AND training) OR (“mentalization-based” AND training) OR “mentalisation-based therapy” OR “mentalization-based therapy” OR “milieu therapy” OR mindfulness OR “music therapy” OR “narrative therapy” OR “play therapy” OR “problem-solving therapy” OR “psychoanalytic therapy” OR “psychological desensitization*” OR “psychological feedback” OR “psychological intervention*” OR “psychological therapy” OR “psychological treatment*” OR psychotherap* OR “reality therapy” OR “sensory feedback” OR “socioenvironmental therapy” OR “STEPPS” OR (“systems training” AND “emotional predictability” AND “problem solving*”)):ab,ti,kw

**# Studytype 🡪 niet gebruikt, in deze database zitten alleen Clinical trials**("clinical trial*” OR “controlled trial*” OR feasib* OR placebo OR random OR randomised OR randomising OR randomized OR randomizing OR randomly OR RCT* OR ((singl* OR doubl* OR trebl* OR tripl*) AND (mask* OR blind* OR dumm*))):ab,ti,kw

**Changes to protocol**

The way both primary outcomes were eventually measured in this NMA, slightly differs from the published PROSPERO file (CRD42020175411). A few adjustments have been made, such as extracting single BPD symptoms from studies if overall BPD severity was not measured. After the PROSPERO protocol was published (CRD42020175411), we also decided to examine how many studies investigated the long-term effects of their treatment. If available, we extracted this data. In contrast to the Prospero registration, we also included studies if they had < 10% non-full BPD-diagnosis patients, or included subthreshold (3 or more BPD-criteria) patients.

**Supplementary Table 2**. Procedure classification of nodes

Based on the expertise from one of the co-authors (AA), each specialised psychotherapy was classified in separate categories. one of the following categories: mentalisation based therapy (MBT), transference-focused therapy (TFP), schema therapy (ST), dialectical behaviour therapy (DBT), cognitive behaviour therapy (CBT), and psychodynamic psychotherapy (PDP). DBT consists of four standard DBT components: individual therapy, group skills training, outside session telephone support, and therapist consultation. Some studies investigated a ‘simplified/stripped’ version of DBT, such as DBT-interpersonal effectiveness (DBT-IE) or DBT-individual therapy (DBT-I) compared to TAU or to another treatment. We merged studies using less than four components of DBT together with studies including all four DBT components, into one DBT category. We tested the influence of this decision in a sensitivity analysis. Based on the classification of a previous meta-analysis by Oud et al. (Oud, Arntz, Hermens, Verhoef, & Kendall, 2018), we categorised structured clinical management (SCM), supervised team management (STM), and client-centered therapy (CCT) all together into the same category (generic treatments for BPD; GT), given that these treatments use a generic approach, not specific for BPD. These treatments often involve BPD-informed clinicians who may be considered as experts and who may have provided a higher level of care than what is typically available in TAU (Finch, Iliakis, Masland, & Choi-Kain, 2019) (see below the criteria for the TAU condition that we perceived as TAU in this study). Community Treatment by Experts (CTBE) was viewed as an optimised variant of TAU (psychotherapy-as-usual provided by trained community experts with experience in treating people with BPD/PDs), and thus constitutes a more stringent comparison condition then TAU (Linehan et al., 2006), which we distinguished as a separate category. Interventions using multiple components from different types of treatments were classified as a category called “mixed interventions” (mixed). Details about the TAU condition were extracted and reported in a predefined table format independently by both assessors (KS & CP). In BPD trials, a common problem is that the nature of TAU conditions are reported inconsistently across studies (Finch et al., 2019). Some studies provided little to no information about the control condition (Carter, Willcox, Lewin, Conrad, & Bendit, 2010), whereas others described their control condition in great detail (Gregory et al., 2008). If a clear description of a TAU condition was lacking, we searched for protocols and trials registrations. If these were not published or when authors still did not provide the wanted information, we categorised conditions as TAU in our NMA, if authors themselves designated a study arm as TAU or if TAU was defined as unspecialised, typical care. Studies delivered in any format (group, individual, or both) were pooled into the same category. In case a modality was used as an adjunct to an existing treatment (telephone-based sessions added to face-to-face sessions) we also classified them into the same category. In an NMA, it is not possible to perform statistical analyses if an RCT investigated a comparison between two types of psychotherapy belonging to the same category (for example two variants of DBT), and therefore these studies were excluded. We did not exclude 3-arm trials comparing two types of psychotherapies belonging to the same category (for example two variants of DBT), as long as their third study arm was classified into a different node. Each category was represented as a separate node into the network. Studies examining the effects of a psychological treatment in comparison with a particular pharmacotherapy condition (that was not part of the TAU condition) were excluded.

**Supplementary Table 3.** Risk of bias assessment

|  |  | **Domain 1** | **Domain 2** | **Domain 3** | | | **Domain 4** | | **Domain 5** | |  | | |
| --- | --- | --- | --- | --- | --- | --- | --- | --- | --- | --- | --- | --- | --- |
|  | **Study** | **Randomization process** | **Deviations from intended interventions** | **Missing outcome data** | | | **Measurement of the outcome (bpd severity)** | **Measurement of the outcome (suicide-related)** | **Selection of the reported result (bpd severity)** | **Selection of the reported result (suicide-related)** | **Overall RoB assessment** | | |
| **1** | Amianto, 2011 | low | some concerns | low | | | low^1^ | high | some concerns | some concerns | high (suicide) | some concerns (bpd)* | |
| **2** | Andreasson, 2016 | low | some concerns | high | | | low | low | high | high | high | | |
| **3** | Andreoli, 2016 | some concerns | some concerns | low | | | not measured | high | not measured | some concerns | high | | |
| **4** | Bateman, 2009 | low | low | low (bpd) | | high (suicide)* | high | low | some concerns | some concerns | high | | |
| **5** | Bellino, 2010 | some concerns | high | high | | | low | low | some concerns | some concerns | high | | |
| **6** | Bohus, 2020 | some concerns | some concerns | low | | | high | high | low | low | high | | |
| **7** | Bozzatello, 2020 | some concerns | high | high | | | high | high | some concerns | some concerns | high | | |
| **8** | Carlyle, 2020 | low | high | low | | | not measured | high | high | some concerns | high | | |
| **9** | Carter, 2010 | low | high | high | | | high | high | some concerns | some concerns | high | | |
| **10** | Clarkin, 2007 | some concerns | some concerns | high | | | high | low | some concerns | some concerns | high | | |
| **11** | Cottraux, 2009 | low | some concerns | high | | | high | high | some concerns | some concerns | high | | |
| **12** | Crawford, 2020 | low | some concerns | high | | | not measured | low | not measured | high | high | | |
| **13** | Davidson, 2006 | low | some concerns | low | | | high | low | low | low | some concerns (suicide) | high (bpd)* | |
| **14** | Dixon-Gordon, 2015 | some concerns | low | low | | | high | not measured | some concerns | not measured | high | | |
| **15** | Doering, 2010 | low | low | low | | | low | high | some concerns | some concerns | high (suicide) | some concerns (bpd)* | |
| **16** | Farrell, 2009 | high^2^ | some concerns | high | | | low | not measured | some concerns | not measured | high | | |
| **17** | Feigenbaum, 2012 | low | some concerns | low | | | high | high | some concerns | some concerns | high | | |
| **18** | Giesen-Bloo, 2006 | some concerns | high | low | | | low | low | high | high | high | | |
| **19** | Gregory, 2008 | low | some concerns | low | | | high | high | some concerns | some concerns | high | | |
| **20** | Herpertz. 2020 | some concerns | low | high | | | high | low | some concerns | some concerns | high | | |
| **21** | Hilden, 2020 | some concerns | high | low | | | high | not measured | high | not measured | high | | |
| **22** | Jorgensen, 2013 | some concerns | some concerns | low | | | high | not measured | some concerns | not measured | high | | |
| **23** | Koons, 2001 | some concerns | some concerns | high | | | low | low | some concerns | some concerns | high | | |
| **24** | Kredlow, 2017a | low | high | high | | | low | not measured | some concerns | not measured | high | | |
| **25** | Laurenssen, 2018 | low^3^ | low^4^ | low (bpd) | high (suicide)* | | low | some concerns | high | some concerns | high | | |
| **26** | Leppänen, 2016 | low | some concerns | high | | | low | low | some concerns | some concerns | high | | |
| **27** | Lin, 2019 | some concerns | high | low | | | high | high | some concerns | some concerns | high | | |
| **28** | Linehan, 1991 | some concerns | high | high | | | not measured | low | some concerns | some concerns | high | | |
| **29** | Linehan, 2006 | high | low | low | | | High | low | some concerns | some concerns | high | | |
| **30** | Majdara, 2019 | some concerns | low | high | | | High | not measured | some concerns | some concerns | high | | |
| **31** | McMain, 2017 | low | low | low | | | High | high | high | some concerns | high | | |
| **32** | McMain, 2009 | low | low | low | | | Low | low | some concerns | high | high (suicide)/ | | some concerns (bpd)* |
| **33** | Philips, 2018 | low | high | high | | | Low | high | low | some concerns | high | | |
| **34** | Pistorello, 2012 | some concerns | low | low | | | Low | low | some concerns | some concerns | some concerns | | |
| **35** | Priebe, 2012 | low | low | high | | | High | not measured | some concerns | not measured | high | | |
| **36** | Reneses, 2013 | some concerns | high | high | | | High | high | some concerns | some concerns | high | | |
| **37** | Soler, 2009 | low | high | low | | | Low | low | some concerns | some concerns | high | | |
| **38** | Turner, 2000 | some concerns | some concerns | low | | | High | high | some concerns | some concerns | high | | |
| **39** | Verheul, 2003 | low | low | low | | | High | high | some concerns | some concerns | high | | |
| **40** | Visintini, 2020 | some concerns | some concerns | low | | | High | high | some concerns | some concerns | high | | |
| **41** | Stanley, 2017 | some concerns | High | high | | | not measured | high | not measured | some concerns | high | | |
| **42** | Walton, 2020 | low | low | low | | | Low | low | some concerns | some concerns | some concerns | | |
| **43** | Weinberg, 2006 | low | high | high | | | not measured | low | some concerns | some concerns | high | | |

NM = not measured

^*^ Both primary outcomes were assessed separately on risk of bias. If findings between both study outcomes showed differences for a particular domain, separate scores were illustrated in Supplementary Table 2

^1^ BPD severity was measured with only one item, possibly resulting in lower levels of sensitivity, making it difficult to detect changes in bpd severity during post-test

^2^ Baseline differences were only compared for outcome variables, not for demographic variables

^3^ Randomization was slightly skewed towards the intervention condition due to availability. Randomization process was conducted according to 2:1 ratio, but participants were still randomly allocated

^4^ Assessment of treatment fidelity was not systematic, but the authors did rate it as acceptable

Results of risk of bias assessment are illustrated in Supplementary Table 3. From the 43 RCTs that were included in the statistical analyses, a total of 22 studies (51.2%) reported an adequate randomisation process. In 13 of the included studies (30.2%), we detected minimal deviations from the intended interventions (domain 2). For BPD severity, low risk of bias due to missing outcome data were reported in 24 (55.8%) of the included studies (domain 3), whereas 22 studies (51.2%) were assessed as low risk bias within this domain for suicidal behavior. Given the nature of psychotherapy trials, blinding was often not possible, which resulted in very few trials with low risk of bias. We rated the fifth domain for most studies as ‘some concerns’, because it required that study protocol or trial registrations were published. After we searched for them, we noticed that these documents are not always published for psychotherapy RCTs. Overall, a total of 17 (39.5%) studies published a protocol study or trial registration. Andreoli et al. (2016) mentioned a research protocol in their paper, we therefore contacted the authors, but we did not receive a response. Protocols and trial registrations of six RCTs were registered prospectively (Bohus et al., 2020; Herpertz et al., 2020; Kredlow et al., 2017; Laurenssen et al., 2018; Philips, Wennberg, Konradsson, & Franck, 2018; Stanley, 2017), whereas 11 were published retrospectively (Andreasson et al., 2016; Bateman & Fonagy, 2009; Bozzatello & Bellino, 2020; Carlyle et al., 2020; Crawford et al., 2020; Davidson et al., 2006; Doering et al., 2010; Hilden et al., 2021; McMain, Guimond, Barnhart, Habinski, & Streiner, 2017; McMain et al., 2009; Walton, Bendit, Baker, Carter, & Lewin, 2020). Eight studies (Andreasson et al., 2016; Carlyle et al., 2020; Crawford et al., 2020; Giesen-Bloo et al., 2006; Hilden et al., 2021; Laurenssen et al., 2018; McMain et al., 2017; McMain et al., 2009) showed deviations from their protocol and were assessed as high risk of bias. If one or more domains of a trial is assessed as high risk of bias, these studies are deemed high risk of bias trials, indicating that the found effects in this study should be cautiously interpreted.

**Supplementary Table 4.** Results of pairwise meta-analyses on the efficacy of BPD severity at post-test. Efficacy was calculated with Standardized Mean Differences (SMD; 95%CI).

|  |  | **Pairwise meta-analyses** | | | | | |
| --- | --- | --- | --- | --- | --- | --- | --- |
| **Efficacy** |  | *N* | SMD | 95%CI | *I*^2^ | 95%CI | Egger |
| CBT vs | MBT | 0 |  |  |  |  |  |
|  | DBT | 1 | -0.29 | -0.72, 0.15 | NA | NA | NA |
|  | GT | 1 | -0.22 | -0.86, 0.41 | NA | NA | NA |
|  | PDP | 0 |  |  |  |  |  |
|  | mixed | 1 | *0.54* | 0.25, 0.83 | NA | NA | NA |
|  | IPT | 0 |  |  |  |  |  |
|  | CTBE | 0 |  |  |  |  |  |
|  | TAU | 2 | -0.47 | -1.95, 1.00 | 90 | NA | NA |
| MBT vs | DBT | 0 |  |  |  |  |  |
|  | GT | 1 | *-0.95* | -1.30, -0.59 | NA | NA | NA |
|  | PDP | 1 | -0.33 | -0.84, 0.17 | NA | NA | NA |
|  | mixed | 0 |  |  |  |  |  |
|  | IPT | 0 |  |  |  |  |  |
|  | CTBE | 1 | -0.07 | -0.47, 0.34 | NA | NA | NA |
|  | TAU | 1 | -0.39 | -1.20, 0.42 | NA | NA | NA |
| ST vs | TFP | 1 | *-0.45* | -0.87, -0.02 | NA | NA | NA |
|  | DBT | 0 |  |  |  |  |  |
|  | GT | 0 |  |  |  |  |  |
|  | PDP | 0 |  |  |  |  |  |
|  | mixed | 0 |  |  |  |  |  |
|  | IPT | 0 |  |  |  |  |  |
|  | CTBE | 0 |  |  |  |  |  |
|  | TAU | 2 | -1.34 | -2.87, 0.18 | 84 | NA | NA |
| TFP vs | DBT | 1 | -0.02 | -0.66, 0.62 | NA | NA | NA |
|  | GT | 0 |  |  |  |  |  |
|  | PDP | 1 | 0.21 | -0.37, 0.80 | NA | NA | NA |
|  | Mixed | 0 |  |  |  |  |  |
|  | IPT | 0 |  |  |  |  |  |
|  | CTBE | 1 | *-0.55* | -0.95, -0.16 | 0 | NA | NA |
|  | TAU | 0 |  |  |  |  |  |
| DBT vs | GT | 1 | *-1.05* | -1.91, -0.18 | NA | NA | NA |
|  | PDP | 4 | -0.20 | -0.61, 0.21 | 75 | 30-91 | 0.62 |
|  | mixed | 2 | 0.04 | -0.23, 0.32 | 0 | NA | NA |
|  | IPT | 0 |  |  |  |  |  |
|  | CTBE | 3 | -0.36 | -0.90, 0.17 | 69 | 0-91 | 0.98 |
|  | TAU | 6 | *-0.35* | -0.58, -0.11 | 0 | 0-75 | 0.17 |
| GT vs | PDP | 1 | 0.0 | -0.68, 0.68 | NA | NA | NA |
|  | mixed | 0 |  |  |  |  |  |
|  | IPT | 0 |  |  |  |  |  |
|  | CTBE | 0 |  |  |  |  |  |
|  | TAU | 0 |  |  |  |  |  |
| PDP vs | mixed | 0 |  |  |  |  |  |
|  | IPT | 0 |  |  |  |  |  |
|  | CTBE | 0 |  |  |  |  |  |
|  | TAU | 3 | *-0.92* | -1.56, -0.28 | 54 | 0-87 | 0.49 |
| mixed vs | IPT | 0 |  |  |  |  |  |
|  | CTBE | 0 |  |  |  |  |  |
|  | TAU | 2 | -0.002 | -0.69, 0.68 | 66 | NA | NA |
| IPT vs | CTBE | 0 |  |  |  |  |  |
|  | TAU | 2 | -0.54 | -1.56, 0.49 | 80 | NA | NA |
| CTBE vs | TAU | 0 |  |  |  |  |  |

* Statistical significant levels of heterogeneity (*p* < 0.05)

NA= not applicable. This cannot be calculated when the number of studies is smaller than 3


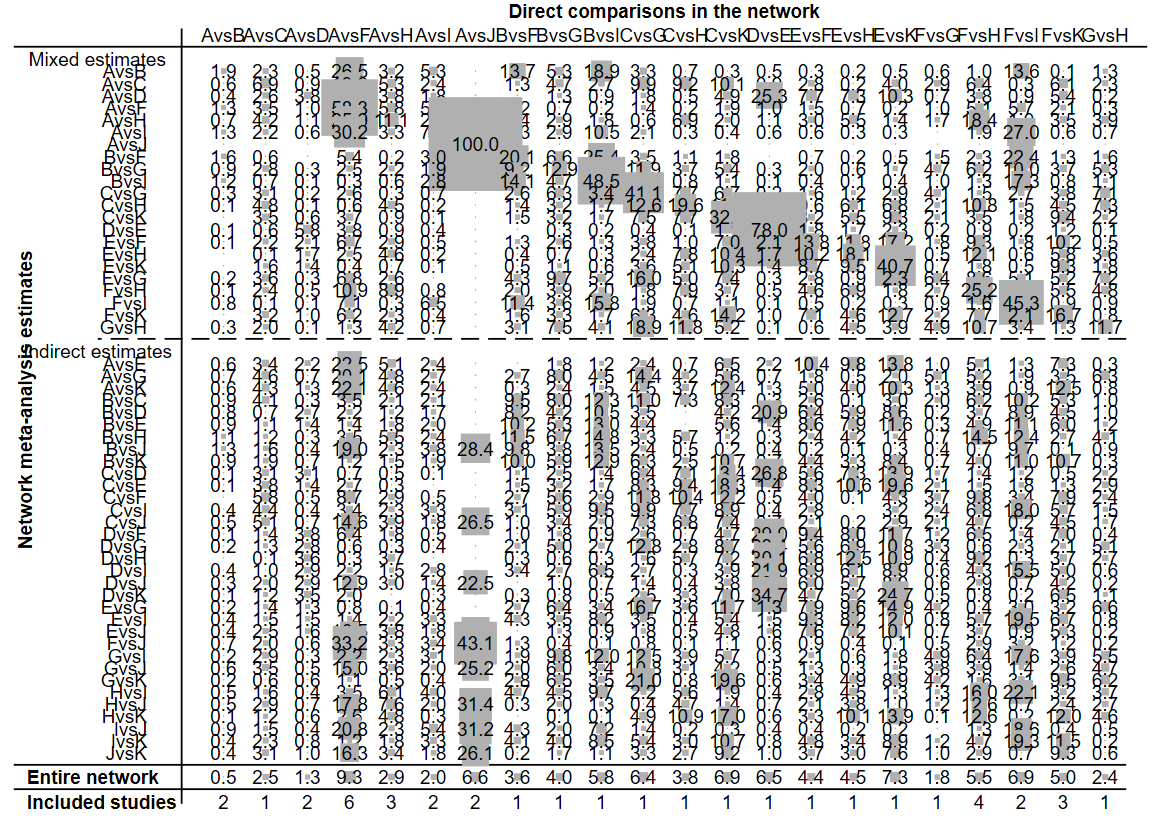
**Supplementary Table 5a.** Contribution plot for BPD severity

**Supplementary Table 5b.** Contribution plot for suicidal behavior

**Supplementary Table 6.** Results of pairwise meta-analysis on the efficacy of suicidal behavior at post-test. Efficacy of pairwise meta-analyses was calculated with Relative Risk (RR; 95%CI). If RRs are larger than 1, its is in favor of the right-column condition when compared to the left-column condition.

| **Suicidal behavior** |  | **Pairwise meta-analyses** | | | | | |
| --- | --- | --- | --- | --- | --- | --- | --- |
|  |  |  |  |  |  |  |  |
| **Efficacy** |  | *N* | RR | 95%CI | *I*^2^ | 95%CI | Egger |
|  |  |  |  |  |  |  |  |
| CBT vs | MBT | 0 |  |  |  |  |  |
|  | ST | 0 |  |  |  |  |  |
|  | DBT | 1 | 1.05 | 0.33, 3.35 | NA | NA | NA |
|  | TFP | 0 |  |  |  |  |  |
|  | GT | 1 | 0.90 | 0.02, 43.39 | NA | NA | NA |
|  | PDP | 0 |  |  |  |  |  |
|  | mixed | 1 | 1.03 | 0.02, 51.45 | NA | NA | NA |
|  | CTBE | 0 |  |  |  |  |  |
|  | TAU | 1 | 0.78 | 0.47, 1.27 | NA | NA | NA |
| MBT vs | ST | 0 |  |  |  |  |  |
|  | DBT | 0 |  |  |  |  |  |
|  | TFP | 0 |  |  |  |  |  |
|  | GT | 2 | 0.36 | 0.04, 2.92 | 87 | NA | NA |
|  | PDP | 0 |  |  |  |  |  |
|  | mixed | 0 |  |  |  |  |  |
|  | CTBE | 0 |  |  |  |  |  |
|  | TAU | 1 | 0.13 | 0.007, 2.41 | NA | NA | NA |
| ST vs | DBT | 0 |  |  |  |  |  |
|  | TFP | 1 | 0.32 | 0.03, 2.94 | NA | NA | NA |
|  | GT | 0 |  |  |  |  |  |
|  | PDP | 0 |  |  |  |  |  |
|  | mixed | 0 |  |  |  |  |  |
|  | CTBE | 0 |  |  |  |  |  |
|  | TAU | 0 |  |  |  |  |  |
| DBT vs | TFP | 1 | 0.79 | 0.40, 1.57 | NA | NA | NA |
|  | GT | 0 |  |  |  |  |  |
|  | PDP | 3 | 0.86 | 0.69, 1.08 | 0 | 0-90 | 0.50 |
|  | mixed | 1 | 2.15 | 0.81, 5.68 | NA | NA | NA |
|  | CTBE | 3 | 1.06 | 0.22, 5.05 | 32 | 0-93 | 0.42 |
|  | TAU | 6 | 0.99 | 0.72, 1.36 | 0 | 0-75 | 0.37 |
| TFP vs | GT | 0 |  |  |  |  |  |
|  | PDP | 1 | 1.04 | 0.59, 1.85 | NA | NA | NA |
|  | mixed | 0 |  |  |  |  |  |
|  | CTBE | 1 | 0.64 | 0.27, 1.51 | NA | NA | NA |
|  | TAU | 0 |  |  |  |  |  |
| GT vs | PDP | 1 | 1.95 | 0.94, 4.02 | NA | NA | NA |
|  | mixed | 0 |  |  |  |  |  |
|  | CTBE | 0 |  |  |  |  |  |
|  | TAU | 0 |  |  |  |  |  |
| PDP vs | mixed | 0 |  |  |  |  |  |
|  | CTBE | 0 |  |  |  |  |  |
|  | TAU | 2 | 0.84 | 0.48, 1.48 | 0 | NA | NA |
| mixed vs | CTBE | 1 | 0.07 | 0.003, 1.76 | NA | NA | NA |
|  | TAU | 3 | 0.83 | 0.58, 1.19 | 0 | 0-90 | 0.66 |
| CTBE vs | TAU | 0 |  |  |  |  |  |

* Statistical significant levels of heterogeneity (*p* < 0.05)

NA= not applicable. This cannot be calculated when the number of studies is smaller than two

**Supplementary Table 7.** Relative effect sizes of efficacy (SMD) for psychotherapies on BPD at post-treatment after conducting a sensitivity analysis by only including studies investigating a full DBT intervention. The diagonal illustrates the different nodes that were examined in this study. Effect sizes are illustrated as SMD with 95%CIs. Data underlined is statistically significant. Comparisons between treatments should be read from left to right, and the estimate is in the cell in common between the column-defining treatment and the row-defining treatment. Negative values indicate that the row-defining intervention is less efficacious than the column-defining intervention. TAU= treatment-as-usual; Mixed= mixed therapeutic techniques; PDP= psychodynamic psychotherapy; IPT= interpersonal psychotherapy; GT= generic treatments for BPD; DBT= dialectical behavior therapy; TFP= transference-focused therapy; ST= schema therapy; MBT= mentalization-based therapy; CBT= cognitive behavior therapy.

| **TAU** |  |  |  |  |  |  |  |  |  |  |  |  |
| --- | --- | --- | --- | --- | --- | --- | --- | --- | --- | --- | --- | --- |
| 0.13 (-0.34, 0.61) | **CTBE** |  |  |  |  |  |  |  |  |  |  |  |
| 0.51 (-0.14, 1.16) | 0.38 (-0.43, 1.18) | **IPT** |  |  |  |  |  |  |  |  |  |  |
| 0.34 (-0.06, 0.74) | 0.20 (-0.35, 0.75) | -0.17 (-0.94, 0.59) | **Mixed** |  |  |  |  |  |  |  |  |  |
| *0.49 (0.12, 0.87)* | 0.36 (-0.13, 0.84) | -0.02 (-0.77, 0.73) | 0.15 (-0.33, 0.64) | **PDP** |  |  |  | | |  |  |  |
| -0.18 (-0.72, 0.37) | -0.31 (-0.92, 0.30) | -0.69 (-1.53, 0.16) | -0.52 (-1.13, 0.10) | *-0.67 (-1.20, -0.14)* | **GT** |  | |  |  | |  |  |
| *0.41 (0.09, 0.72)* | 0.27 (-0.13, 0.67) | -0.10 (-0.83, 0.62) | 0.07 (-0.34, 0.47) | -0.09 (-0.43, 0.26) | *0.58 (0.05, 1.11)* | **DBT** | |  |  |  |  |  |
| *0.57 (0.02, 1.13)* | 0.44 (-0.10, 0.98) | 0.06 (-0.79, 0.92) | 0.23 (-0.40, 0.87) | 0.08 (-0.48, 0.64) | *0.75 (0.05, 1.45)* | 0.17 (-0.36, 0.70) | | **TFP** |  |  |  |  |
| *1.13 (0.53,1.74)* | *1.00 (0.31, 1.69)* | 0.62 (-0.26, 1.51) | *0.79 (0.09, 1.50)* | 0.64 (-0.02, 1.30) | *1.31 (0.53, 2.09)* | *0.73 (0.09, 1.36)* | | 0.56 (-0.05, 1.17) | **ST** | |  |  |
| *0.56 (0.05, 1.07)* | 0.43 (-0.10, 0.95) | 0.05 (-0.77, 0.88) | 0.22 (-0.37, 0.82) | 0.07 (-0.43, 0.56) | *0.74 (0.20 1.28)* | 0.16 (-0.34, 0.65) | | -0.01 (-0.67, 0.65) | -0.57 (-1.32, 0.17) | | **MBT** |  |
| 0.06 (-0.41, 0.54) | -0.07 (-0.69, 0.55) | -0.45 (-1.25, 0.36) | -0.27 (-0.78, 0.23) | -0.43 (-0.98, 0.12) | 0.24 (-0.36, 0.84) | -0.34 (-0.85, 0.17) | | -0.51 (-1.20, 0.19) | *-1.07 (-1.81, -0.32)* | | -0.50 (-1.13, 0.14) | **CBT** |

**Efficacy at post-test (SMD with 95%CI)**

| **TAU** |  | | | |  |  |  |  |  |  |
| --- | --- | --- | --- | --- | --- | --- | --- | --- | --- | --- |
| 1.00 (0.28, 3.58) | **CTBE** |  | | |  |  |  |  |  |  |
| 1.72 (0.69, 4.30) | 1.72 (0.43, 6.93) | **Mixed** |  | | |  |  |  |  |  |
| 1.39 (0.40, 4.84) | 1.39 (0.38, 5.08) | 0.81 (0.20, 3.17) | **PDP** |  | |  |  |  |  |  |
| 2.36 (0.18, 31.37) | 2.36 (0.14, 40.15) | 1.37 (0.09, 21.34) | 1.71 (0.10, 29.28) | **GT** |  |  |  |  |  |  |
| 1.23 (0.55, 2.76) | 1.23 (0.44, 3.46) | 0.72 (0.27, 1.92) | 0.89 (0.34, 2.34) | 0.52 (0.04, 7.59) | **DBT** |  |  | | |  |
| 1.30 (0.36, 4.71) | 1.30 (0.43, 3.91) | 0.75 (0.18, 3.08) | 0.93 (0.31, 2.85) | 0.55 (0.03, 9.51) | 1.05 (0.38, 2.93) | **TFP** |  |  |  |  |
| 4.07 (0.25, 67.41) | 4.07 (0.27, 62.11) | 2.37 (0.14, 41.43) | 2.94 (0.19, 45.04) | 1.72 (0.04, 76.11) | 3.30 (0.22, 48.87) | 3.14 (0.26, 37.97) | **ST** | |  | |
| 5.04 (0.40, 62.88) | 5.04 (0.30, 83.73) | 2.93 (0.21, 41.83) | 3.64 (0.22, 58.98) | 2.13 (0.73, 6.21) | 4.09 (0.30, 55.83) | 3.89 (0.23, 64.54) | 1.24 (0.03, 52.88) | | **MBT** |  |
| 1.32 (0.49, 3.51) | 1.31 (0.30, 5.73) | 0.76 (0.22, 2.63) | 0.95 (0.23, 4.00) | 0.56 (0.04, 7.70) | 1.07 (0.37, 3.11) | 1.01 (0.23, 4.45) | 0.32 (0.02, 5.85) | | 0.26 (0.02, 3.42) | **CBT** |

**Supplementary Table 8.** Sensitivity analysis only including studies purely measuring suicidal behavior (suicide attempt and/or death by suicide) reporting Risk Ratios (RRs) for psychotherapies on suicidal behavior post-test according to network meta-analysis. The diagonal illustrates the different nodes that were examined in this study. Data in bold are statistically significant. Comparisons between treatments should be read from left to right, and the estimate is in the cell in common between the column-defining treatment and the row-defining treatment. To obtain RRs for comparisons in the opposite direction, reciprocals should be taken. Data bold and underlined are statistically significant. TAU= treatment-as-usual; MBT= mentalization-based therapy; TFP= transference-focused therapy; CTBE= community treatment by experts; Mixed= mixed therapeutic techniques; PDP= psychodynamic psychotherapy; GT= generic treatments for BPD; DBT= dialectical behavior therapy; CBT= cognitive behavior therapy; ST = schema therapy.

**Efficacy at post-test (RR with 95%CI)**

**Supplementary Table 9.** Relative effect sizes of efficacy (SMD) for psychotherapies on BPD at post-treatment after conducting a sensitivity analysis by only including studies measuring overall BPD symptom severity. The diagonal illustrates the different nodes that were examined in this study. Effect sizes are illustrated as SMD with 95%CIs. Data underlined is statistically significant. Comparisons between treatments should be read from left to right, and the estimate is in the cell in common between the column-defining treatment and the row-defining treatment. Negative values indicate that the row-defining intervention is less efficacious than the column-defining intervention. TAU= treatment-as-usual; Mixed= mixed therapeutic techniques; PDP= psychodynamic psychotherapy; IPT= interpersonal psychotherapy; GT= generic treatments for BPD; DBT= dialectical behavior therapy; TFP= transference-focused therapy; ST= schema therapy; MBT= mentalization-based therapy; CBT= cognitive behavior therapy.

| **TAU** |  |  |  |  |  |  |  |  |  |  |  |  |
| --- | --- | --- | --- | --- | --- | --- | --- | --- | --- | --- | --- | --- |
| 0.12 (-0.62, 0.86) | **CTBE** |  |  |  |  |  |  |  |  |  |  |  |
| 0.52 (-0.24, 1.28) | 0.40 (-0.66, 1.46) | **IPT** |  |  |  |  |  |  |  |  |  |  |
| *0.80 (0.17, 1.44)* | 0.69 (-0.23, 1.61) | 0.28 (-0.71, 1.27) | **Mixed** |  |  |  |  |  |  |  |  |  |
| *0.51 (0.04, 0.97)* | 0.39 (-0.38, 1.15) | -0.02 (-0.90, 0.87) | -0.30 (-1.02, 0.42) | **PDP** |  |  | |  | | |  |  |
| 0.51 (-0.68, 1.70) | 0.39 (-0.95, 1.72) | -0.02 (-1.43, 1.39) | -0.30 (-1.61, 1.01) | -0.0 (-1.10, 1.10) | **GT** |  |  | |  |  |  |  |
| *0.65 (0.25, 1.06)* | 0.53 (-0.18, 1.25) | 0.13 (-0.73, 0.99) | -0.15 (-0.77, 0.47) | 0.15 (-0.29, 0.58) | 0.15 (-1.03, 1.32) | **DBT** |  |  |  |  | |  |
| 0.72 (-0.17, 1.62) | 0.61 (-0.20, 1.41) | 0.20 (-0.97, 1.38) | -0.08 (-1.15, 0.99) | 0.22 (-0.73, 1.17) | 0.22 (-1.23, 1.67) | 0.07 (-0.85, 0.99) | **TFP** | |  |  |  |  |
| *1.22 (0.48, 1.97)* | *1.11 (0.22, 2.00)* | 0.70 (-0.36, 1.77) | 0.42 (-0.55, 1.39) | 0.72 (-0.13, 1.57) | 0.72 (-0.67, 2.10) | 0.57 (-0.24, 1.39) | 0.50 (-0.31, 1.31) | | **ST** |  |  |  |
| 0.47 (-0.23, 1.17) | 0.35 (-0.38, 1.09) | -0.05 (-1.08, 0.98) | -0.33 (-1.24, 0.57) | -0.03 (-0.73, 0.66) | -0.03 (-1.33, 1.26) | -0.18 (-0.89, 0.53) | -0.25 (-1.24, 0.73) | | -0.75 (-1.70, 0.20) | **MBT** | |  |
| *0.74 (0.07, 1.41)* | 0.62 (-0.32, 1.56) | 0.22 (-0.80, 1.23) | -0.06 (-0.75, 0.62) | 0.23 (-0.51, 0.98) | 0.23 (-1.09, 1.56) | 0.09 (-0.56, 0.74) | 0.02 (-1.07, 1.10) | | -0.48 (-1.47, 0.50) | 0.27 (-0.66, 1.19) | | **CBT** |

**Efficacy at post-test (SMD with 95%CI)**

**Supplementary Table 10.**

Relative effect sizes of efficacy (SMD) for psychotherapies on BPD at post-treatment after conducting a sensitivity analysis on BPD severity by only including studies with a combined format (individual + group sessions). The diagonal illustrates the different nodes that were examined in this study. Effect sizes are illustrated as SMD with 95%CIs. Data underlined is statistically significant. Comparisons between treatments should be read from left to right, and the estimate is in the cell in common between the column-defining treatment and the row-defining treatment. Negative values indicate that the row-defining intervention is less efficacious than the column-defining intervention. TAU= treatment-as-usual; Mixed= mixed therapeutic techniques; PDP= psychodynamic psychotherapy; GT= generic treatments; DBT= dialectical behavior therapy; TFP= transference-focused therapy; MBT= mentalization-based therapy; CBT= cognitive behavior therapy.

| **TAU** |  |  |  |  |  |  |  |  |  |  |
| --- | --- | --- | --- | --- | --- | --- | --- | --- | --- | --- |
| -0.08 (-0.43, 0.27) | **CTBE** |  |  |  |  |  |  |  |  |  |
| 0.15 (-0.14, 0.43) | 0.23 (-0.13, 0.59) | **Mixed** |  |  |  |  |  |  |  |  |
| 0.12 (-0.19, 0.43) | 0.20 (-0.11, 0.52) | -0.03 (-0.34, 0.28) | **PDP** |  |  | |  | |  |  |
| *-0.77 (-1.25, -0.29)* | *-0.69 (-1.14, -0.25)* | *-0.92 (-1.42, -0.43)* | *-0.89 (-1.34, -0.45)* | **GT** |  |  | |  |  |  |
| 0.18 (-0.06, 0.42) | 0.26 (-0.00, 0.53) | 0.03 (-0.22. 0.28) | 0.06 (-0.14, 0.25) | *0.95 (0.52, 1.38)* | **DBT** |  |  |  | |  |
| 0.03 (-0.55, 0.61) | 0.11 (-0.48, 0.70) | -0.12 (-0.71, 0.47) | -0.09 (-0.62, 0.44) | *0.80 (0.13, 1.48)* | -0.15 (-0.68, 0.39) | **TFP** | |  |  |  |
| 0.19 (-0.19, 0.57) | 0.27 (-0.06, 0.60) | 0.04 (-0.36, 0.44) | 0.07 (-0.27, 0.41) | *0.96 (0.62, 1.30)* | 0.01 (-0.31, 0.33) | 0.16 (-0.45, 0.77) | | **MBT** | |  |
| -0.39 (-0.80, 0.02) | -0.31 (-0.77, 0.15) | *-0.54 (-0.83, -0.25)* | *-0.51 (-0.94, -0.08)* | 0.38 (-0.19, 0.96) | *-0.57 (-0.95, -0.19)* | -0.42 (-1.08, 0.24) | | *-0.58*  *(-1.08, -0.08)* | | **CBT** |

**Efficacy at post-test (SMD with 95%CI)**

**Supplementary Table 11.** Sensitivity analyses on the efficacy of psychotherapies for BPD severity, by only including studies measuring overall bpd symptom severity- and studies investigating a full DBT intervention

| **Contrast** | | **Pairwise meta-analyses** | | | | | |  | **Contrast** | | **Pairwise meta-analyses** | | | | | |
| --- | --- | --- | --- | --- | --- | --- | --- | --- | --- | --- | --- | --- | --- | --- | --- | --- |
|  |  | overall BPD symptom severity | | | | | |  |  |  | full DBT intervention | | | | | |
|  |  | *N* | *SMD* | *95%CI* | *I^2^* | *95%CI* | *Egger* |  |  |  | *N* | *SMD* | *95%CI* | *I^2^* | *95%CI* | *Egger* |
| CBT vs | MBT | 0 |  |  |  |  |  |  | CBT vs | MBT | 0 |  |  |  |  |  |
|  | ST | 0 |  |  |  |  |  |  |  | ST | 0 |  |  |  |  |  |
|  | TFP | 0 |  |  |  |  |  |  |  | TFP | 0 |  |  |  |  |  |
|  | DBT | 1 | -0.29 | -0.72, 0.15 | NA | NA | NA |  |  | DBT | 0 |  |  |  |  |  |
|  | GT | 0 |  |  |  |  |  |  |  | GT | 1 | -0.22 | -0.86, 0.41 | NA | NA | NA |
|  | PDP | 0 |  |  |  |  |  |  |  | PDP | 0 |  |  |  |  |  |
|  | mixed | 1 | *0.54* | 0.25, 0.83 | NA | NA | NA |  |  | mixed | 1 | *0.54* | 0.25, 0.83 | NA | NA | NA |
|  | IPT | 0 |  |  |  |  |  |  |  | IPT | 0 |  |  |  |  |  |
|  | CTBE | 0 |  |  |  |  |  |  |  | CTBE | 0 |  |  |  |  |  |
|  | TAU | 1 | *-1.28* | -2.14, -0.42 | NA | NA | NA |  |  | TAU | 2 | -0.47 | -1.95, 1.00 | 90 | NA | NA |
| MBT vs | ST | 0 |  |  |  |  |  |  | MBT vs | ST | 0 |  |  |  |  |  |
|  | TFP | 0 |  |  |  |  |  |  |  | TFP | 0 |  |  |  |  |  |
|  | DBT | 0 |  |  |  |  |  |  |  | DBT | 0 |  |  |  |  |  |
|  | GT | 0 |  |  |  |  |  |  |  | GT | 1 | *-0.95* | -1.30, -0.59 | NA | NA | NA |
|  | PDP | 1 | -0.33 | -0.84, 0.17 | NA | NA | NA |  |  | PDP | 1 | -0.33 | -0.84, 0.17 | NA | NA | NA |
|  | mixed | 0 |  |  |  |  |  |  |  | mixed | 0 |  |  |  |  |  |
|  | IPT | 0 |  |  |  |  |  |  |  | IPT | 0 |  |  |  |  |  |
|  | CTBE | 1 | -0.07 | -0.47, 0.39 | NA | NA | NA |  |  | CTBE | 1 | -0.07 | -0.47, 0.33 | NA | NA | NA |
|  | TAU | 1 | -0.39 | -1.20, 0.42 | NA | NA | NA |  |  | TAU | 1 | -0.39 | -1.20, 0.42 | NA | NA | NA |
| ST vs | TFP | 1 | *-0.45* | -0.87, -0.02 | NA | NA | NA |  | ST vs | TFP | 1 | *-0.45* | -0.87, -0.02 | NA | NA | NA |
|  | DBT | 0 |  |  |  |  |  |  |  | DBT | 0 |  |  |  |  |  |
|  | GT | 0 |  |  |  |  |  |  |  | GT | 0 |  |  |  |  |  |
|  | PDP | 0 |  |  |  |  |  |  |  | PDP | 0 |  |  |  |  |  |
|  | mixed | 0 |  |  |  |  |  |  |  | mixed | 0 |  |  |  |  |  |
|  | IPT | 0 |  |  |  |  |  |  |  | IPT | 0 |  |  |  |  |  |
|  | CTBE | 0 |  |  |  |  |  |  |  | CTBE | 0 |  |  |  |  |  |
|  | TAU | 2 | -1.34 | -2.86, 0.18 | 84 | NA | NA |  |  | TAU | 2 | -1.34 | -2.86, 0.18 | 84 | NA | NA |
| TFP vs | DBT | 0 |  |  |  |  |  |  | TFP vs | DBT | 1 | -0.02 | -0.66, 0.62 | NA | NA | NA |
|  | GT | 0 |  |  |  |  |  |  |  | GT | 0 |  |  |  |  |  |
|  | PDP | 0 |  |  |  |  |  |  |  | PDP | 1 | 0.21 | -0.37, 0.80 | NA | NA | NA |
|  | mixed | 0 |  |  |  |  |  |  |  | mixed | 0 |  |  |  |  |  |
|  | IPT | 0 |  |  |  |  |  |  |  | IPT | 0 |  |  |  |  |  |
|  | CTBE | 1 | *-0.55* | -0.95, -0.16 | NA | NA | NA |  |  | CTBE | 1 | *-0.55* | -0.95, -0.16 | NA | NA | NA |
|  | TAU | 0 |  |  |  |  |  |  |  | TAU | 0 |  |  |  |  |  |
| DBT vs | GT | 0 |  |  |  |  |  |  | DBT vs | GT | 1 | *-1.05* | -1.91, -0.18 | NA | NA | NA |
|  | PDP | 3 | -0.31 | -0.79, 0.17 | 81 | 40-94 | 0.05 |  |  | PDP | 3 | -0.02 | -0.22, 0.19 | 0 | 0-90 | 0.07 |
|  | mixed | 1 | 0.01 | -0.36, 0.39 | NA | NA | NA |  |  | mixed | 2 | 0.04 | -0.23, 0.32 | 0 | NA | NA |
|  | IPT | 0 |  |  |  |  |  |  |  | IPT | 0 |  |  |  |  |  |
|  | CTBE | 1 | -0.91 | -1.43, 0.39 | NA | NA | NA |  |  | CTBE | 3 | -0.37 | -0.90, 0.17 | 69 | 0-91 | 0.99 |
|  | TAU | 4 | *-0.47* | -0.76, -0.18 | 0 | 0-85 | 0.20 |  |  | TAU | 4 | -0.23 | -0.52,0.06 | 0 | 0-85 | 0.78 |
| GT vs | PDP | 1 | 0.0 | 0.68, -0.68 | NA | NA | NA |  | GT vs | PDP | 1 | 0.0 | 0.68. -0.68 | NA | NA | NA |
|  | mixed | 0 |  |  |  |  |  |  |  | mixed | 0 |  |  |  |  |  |
|  | IPT | 0 |  |  |  |  |  |  |  | IPT | 0 |  |  |  |  |  |
|  | CTBE | 0 |  |  |  |  |  |  |  | CTBE | 0 |  |  |  |  |  |
|  | TAU | 0 |  |  |  |  |  |  |  | TAU | 0 |  |  |  |  |  |
| PDP vs | mixed | 0 |  |  |  |  |  |  | PDP vs | mixed | 0 |  |  |  |  |  |
|  | IPT | 0 |  |  |  |  |  |  |  | IPT | 0 |  |  |  |  |  |
|  | CTBE | 0 |  |  |  |  |  |  |  | CTBE | 0 |  |  |  |  |  |
|  | TAU | 3 | *-0.92* | -1.56, -0.28 | 54 | 0-87 | 0.49 |  |  | TAU | 3 | *-0.92* | -1.56, -0.28 | 54 | 0-87 | 0.49 |
| mixed vs | IPT | 0 |  |  |  |  |  |  | mixed vs | IPT | 0 |  |  |  |  |  |
|  | CTBE | 0 |  |  |  |  |  |  |  | CTBE | 0 |  |  |  |  |  |
|  | TAU | 1 | -0.35 | -0.93, 0.22 | NA | NA | NA |  |  | TAU | 2 | -0.002 | -0.69, 0.68 | 66 | NA | NA |
| IPT vs | CTBE | 0 |  |  |  |  |  |  | IPT vs | CTBE | 0 |  |  |  |  |  |
|  | TAU | 2 | -0.54 | -1.56, 0.49 | 80 | NA | NA |  |  | TAU | 2 | -0.54 | -1.56, 0.49 | 80 | NA | NA |
| CTBE vs | TAU | 0 |  |  |  |  |  |  | CTBE vs | TAU | 0 |  |  |  |  |  |

NA=not applicable. This cannot be calculated when the number of studies is smaller than 3

**Supplementary Table 12.** Sensitivity analysis on suicidal behavior only including studies measuring purely suicidal behavior (death by suicide- and suicide attempts)

| **Contrast** |  | **Pairwise meta-analyses** | | | |  |  |
| --- | --- | --- | --- | --- | --- | --- | --- |
|  |  |  |  |  |  |  |  |
|  |  |  |  |  |  |  |  |
|  |  | *N* | RR | 95%CI | *I*^2^ | 95%CI | Egger |
| CBT vs | MBT | 0 |  |  |  |  |  |
|  | ST | 0 |  |  |  |  |  |
|  | DBT | 1 | 1.05 | 0.33, 3.35 | NA | NA | NA |
|  | TFP | 0 |  |  |  |  |  |
|  | GT | 1 | 0.90 | 0.02, 43.39 | NA | NA | NA |
|  | PDP | 0 |  |  |  |  |  |
|  | mixed | 1 | 1.03 | 0.02, 51.45 | NA | NA | NA |
|  | CTBE | 0 |  |  |  |  |  |
|  | TAU | 1 | 0.78 | 0.47, 1.27 | NA | NA | NA |
| MBT vs | ST | 0 |  |  |  |  |  |
|  | DBT | 0 |  |  |  |  |  |
|  | TFP | 0 |  |  |  |  |  |
|  | GT | 2 | 0.36 | 0.04, 2.92 | 87 | NA | NA |
|  | PDP | 0 |  |  |  |  |  |
|  | mixed | 0 |  |  |  |  |  |
|  | CTBE | 0 |  |  |  |  |  |
|  | TAU | 1 | 0.13 | 0.007, 2.41 | NA | NA | NA |
| ST vs | DBT | 0 |  |  |  |  |  |
|  | TFP | 1 | 0.32 | 0.03, 2.94 | NA | NA | NA |
|  | GT | 0 |  |  |  |  |  |
|  | PDP | 0 |  |  |  |  |  |
|  | mixed | 0 |  |  |  |  |  |
|  | CTBE | 0 |  |  |  |  |  |
|  | TAU | 0 |  |  |  |  |  |
| DBT vs | TFP | 1 | 0.79 | 0.40, 1.57 | NA | NA | NA |
|  | GT | 0 |  |  |  |  |  |
|  | PDP | 2 | 1.00 | 0.56, 1.78 | 0 | NA | NA |
|  | mixed | 1 | 2.15 | 0.81, 5.68 | NA | NA | NA |
|  | CTBE | 3 | 1.06 | 0.22, 5.05 | 32 | 0-93 | 0.42 |
|  | TAU | 5 | 0.55 | 0.25, 1.23 | 0 | 0-79 | 0.37 |
| TFP vs | GT | 0 |  |  |  |  |  |
|  | PDP | 1 | 1.04 | 0.59, 1.85 | NA | NA | NA |
|  | mixed | 0 |  |  |  |  |  |
|  | CTBE | 1 | 0.64 | 0.27, 1.51 | NA | NA | NA |
|  | TAU | 0 |  |  |  |  |  |
| GT vs | PDP | 0 |  |  |  |  |  |
|  | mixed | 0 |  |  |  |  |  |
|  | CTBE | 0 |  |  |  |  |  |
|  | TAU | 0 |  |  |  |  |  |
| PDP vs | mixed | 0 |  |  |  |  |  |
|  | CTBE | 0 |  |  |  |  |  |
|  | TAU | 0 |  |  |  |  |  |
| mixed vs | CTBE | 1 | 0.07 | 0.003, 1.76 | NA | NA | NA |
|  | TAU | 2 | 0.90 | 0.61, 1.33 | 0 | NA | NA |
| CTBE vs | TAU | 0 |  |  |  |  |  |

NA=not applicable. This cannot be calculated when the number of studies is smaller than 3

**Supplementary Table 13.** Sensitivity analysis on BPD severity only including studies using a combined format (individual + group therapy sessions)

| **Contrast** |  | **Pairwise meta-analyses** | | | |  |  |
| --- | --- | --- | --- | --- | --- | --- | --- |
|  |  |  |  |  |  |  |  |
|  |  |  |  |  |  |  |  |
|  |  | *N* | *SMD* | 95%CI | *I*^2^ | 95%CI | Egger |
| CBT vs | MBT |  |  |  |  |  |  |
|  | DBT |  |  |  |  |  |  |
|  | TFP |  |  |  |  |  |  |
|  | GT |  |  |  |  |  |  |
|  | PDP |  |  |  |  |  |  |
|  | mixed | 1 | *0.54* | 0.25, 0.83 | NA | NA | NA |
|  | CTBE |  |  |  |  |  |  |
|  | TAU |  |  |  |  |  |  |
| MBT vs | DBT |  |  |  |  |  |  |
|  | TFP |  |  |  |  |  |  |
|  | GT | 1 | *-0.95* | -1.30, -0.59 | NA | NA | NA |
|  | PDP | 1 | -0.33 | -0.84, 0.17 | NA | NA | NA |
|  | mixed |  |  |  |  |  |  |
|  | CTBE | 1 | -0.07 | -0.47, 0.39 | NA | NA | NA |
|  | TAU | 1 | -0.39 | -1.20, 0.42 | NA | NA | NA |
| DBT vs | TFP | 1 | 0.02 | -0.62, 0.66 | NA | NA | NA |
|  | GT | 1 | *-1.05* | -1.91, -0.18 | NA | NA | NA |
|  | PDP | 3 | -0.02 | -0.22, 1.18 | 0 | 0-90 | 0.076 |
|  | mixed | 2 | 0.04 | -0.23, 0.32 | 0 | NA | NA |
|  | CTBE | 3 | -0.37 | -0.90, 0.17 | 69 | 0-91 | 0.99 |
|  | TAU | 4 | -0.23 | -0.52, 0.06 | 0 | 0-85 | 0.78 |
| TFP vs | GT |  |  |  |  |  |  |
|  | PDP | 1 | 0.21 | -0.37, 0.80 | NA | NA | NA |
|  | mixed |  |  |  |  |  |  |
|  | CTBE |  |  |  |  |  |  |
|  | TAU |  |  |  |  |  |  |
| GT vs | PDP |  |  |  |  |  |  |
|  | mixed |  |  |  |  |  |  |
|  | CTBE |  |  |  |  |  |  |
|  | TAU |  |  |  |  |  |  |
| PDP vs | mixed |  |  |  |  |  |  |
|  | CTBE |  |  |  |  |  |  |
|  | TAU |  |  |  |  |  |  |
| mixed vs | CTBE |  |  |  |  |  |  |
|  | TAU | 2 | -0.002 | -0.69, 0.68 | 66 | NA | NA |
| CTBE vs | TAU |  |  |  |  |  |  |

NA=not applicable. This cannot be calculated when the number of studies is smaller than 3

**Supplementary Figure 1a.** Inconsistency Factors for the Network


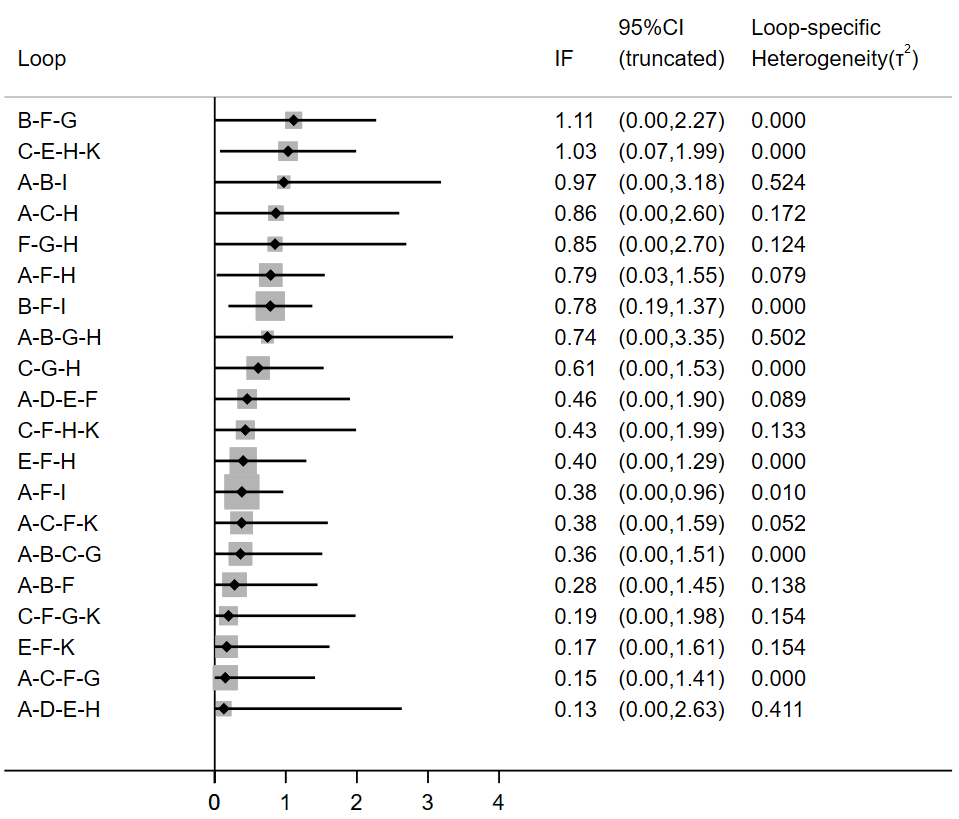


a=treatment-as-usual (TAU); b=cognitive behavioral therapy (CBT); c= mentalization-based therapy (MBT); d=schema therapy (ST); e=transference-focused therapy (TFP); f=dialectical behavior therapy (DBT); g=generic treatments for BPD (GT); h=psychodynamic psychotherapy (PDP); i=mixed approaches/therapeutic techniques (mixed); j= interpersonal psychotherapy (IPT); k=community treatment by experts (CTBE)

**Supplementary Figure 1b.** Inconsistency Factor for the Network


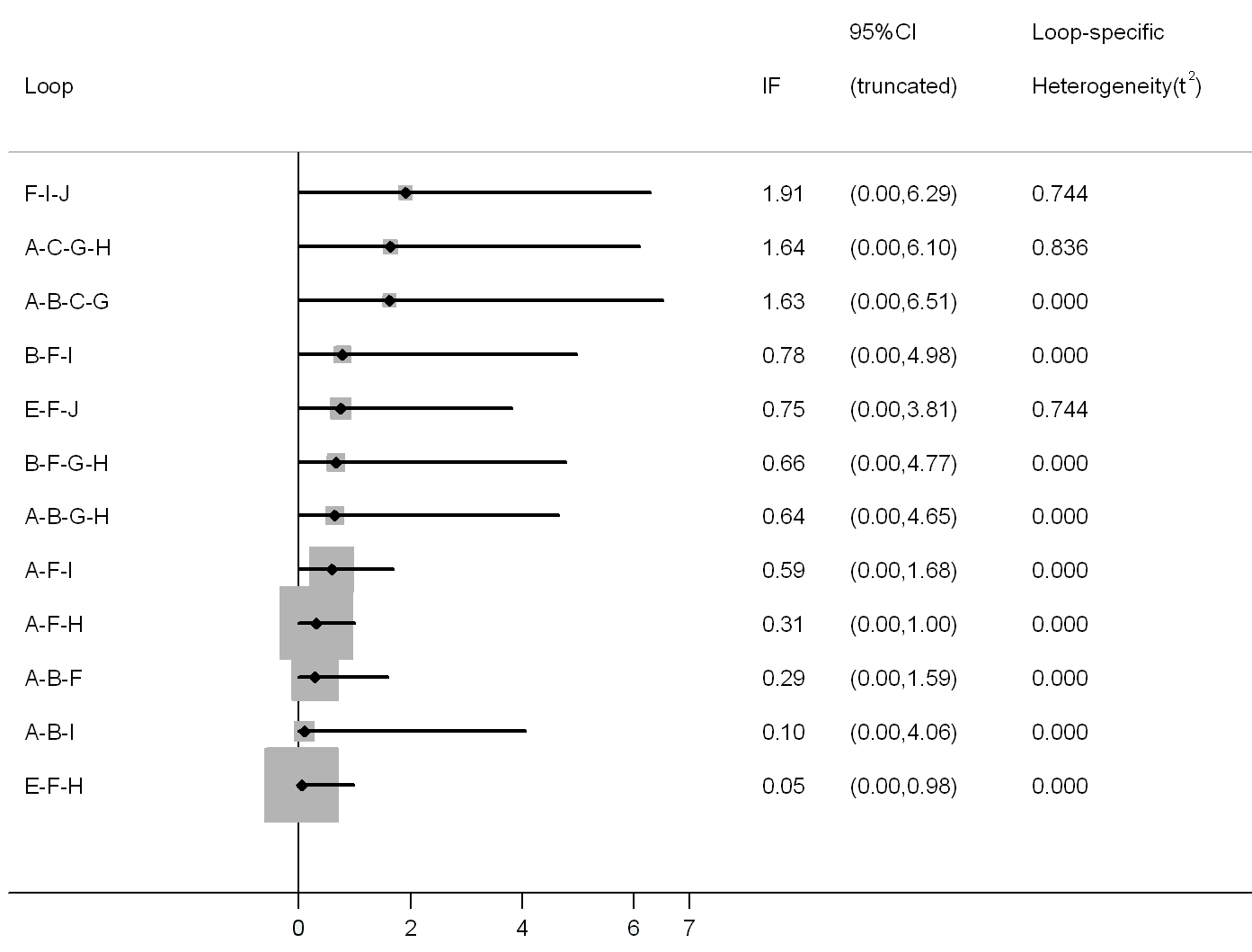


a=treatment-as-usual (TAU); b=cognitive behavioral therapy (CBT); c= mentalization-based therapy (MBT); d=schema therapy (ST); e=transference-focused therapy (TFP); f=dialectical behavior therapy (DBT); g=generic treatments for BPD (GT); h=psychodynamic psychotherapy (PDP); i=mixed approaches/therapeutic techniques (mixed); j=community treatment by experts (CTBE)

**Supplementary Figure 2.** The red line represents the null hypothesis that the study-specific effect sizes do not differ from the respective comparison-specific pooled estimates. Each black dot corresponds to a comparison. A comparison-adjusted funnel plot was conducted for each primary outcome (bpd symptom severity and suicidal behavior), and study drop-out (for any reason)


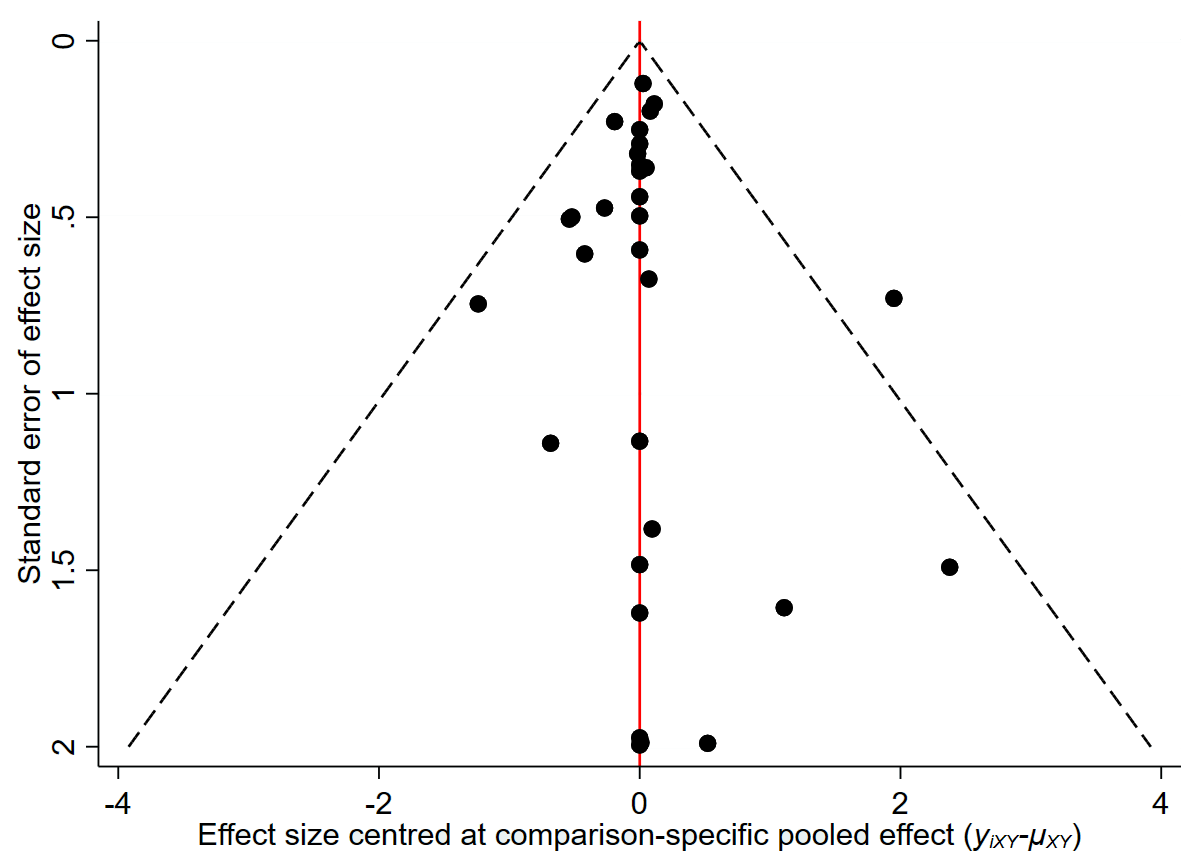
**a. BPD symptom severity b. Suicidal behavior**

**
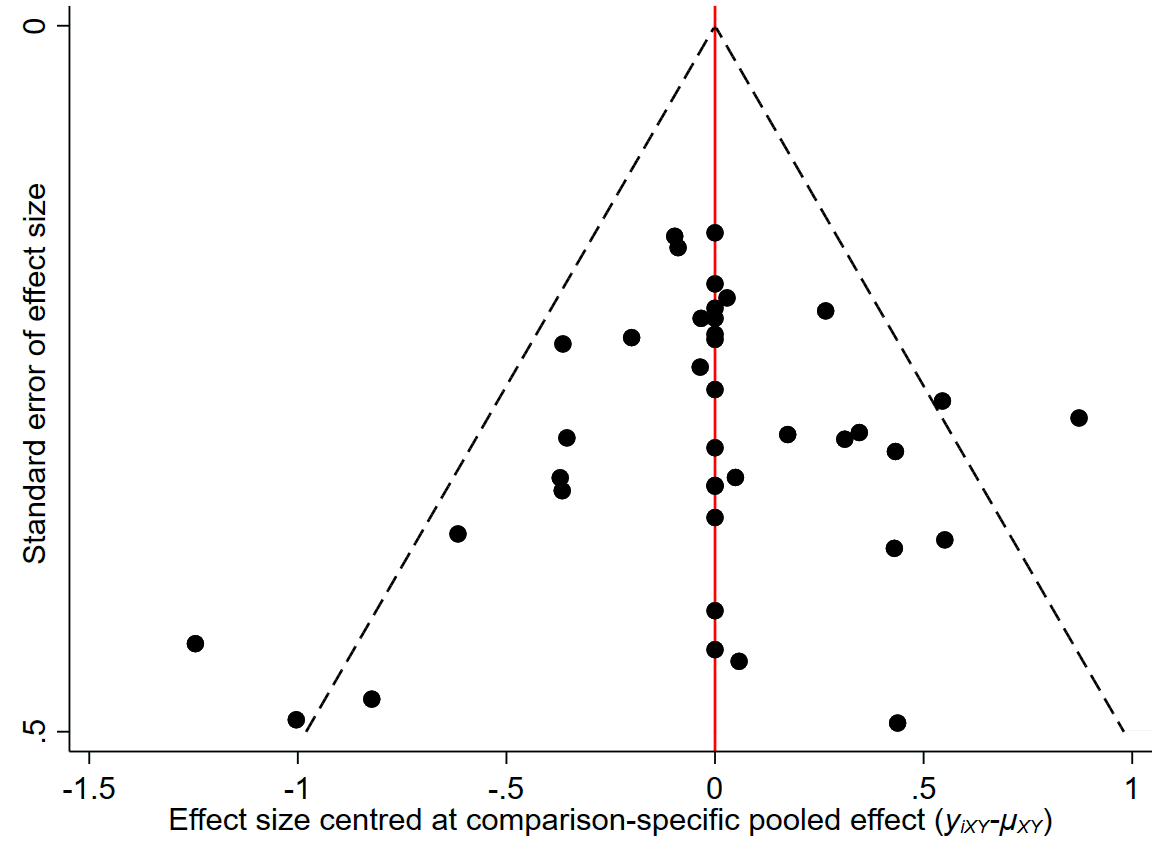
**

**Control conditions more effective**

**Active treatments more effective**

**Active treatments more effective**

**Control conditions more effective**

**c. Study-dropout**


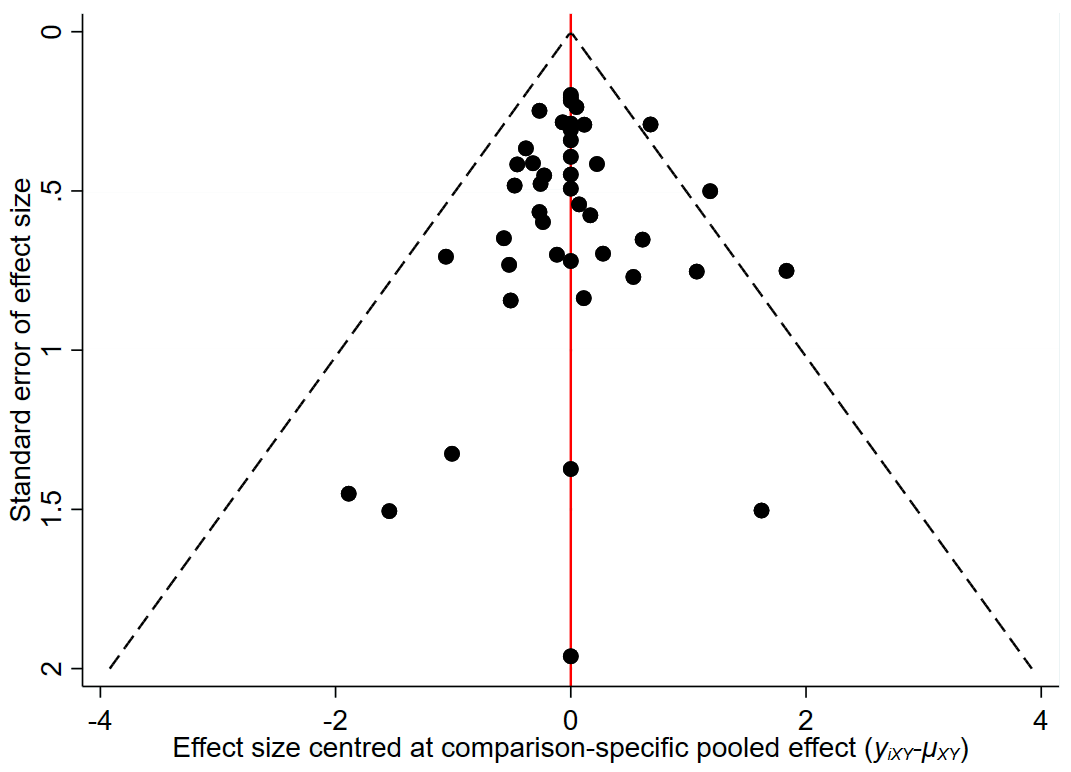

Supplement: Supplementary file 1 [file S0033291723000685sup001.docx]
